# S100A9 as a potential novel target for experimental autoimmune cystitis and interstitial cystitis/bladder pain syndrome

**DOI:** 10.1186/s40364-025-00763-5

**Published:** 2025-05-09

**Authors:** Jiang Zhao, Mi Zhou, Chengfei Yang, Yang-Wuyue Liu, Teng Yang, Bishao Sun, Benyi Li, Ji Zheng, Shuangshuang Dai, Zhenxing Yang, Xiangwei Wang

**Affiliations:** 1 Department of Urology, Guangdong Provincial Key Laboratory of Autophagy and Major Chronic Non-Communicable Diseases, Affiliated Hospital of Guangdong Medical University, Zhanjiang, 524001 PR China; 2 Department of Urology, Second Affiliated Hospital, Army Military Medical University, Chongqing, 400037 PR China; 3 Department of Urology, The University of Kansas Medical Center, Kansas City, KS 66160 USA; 4 Department of Biochemistry and Molecular Biology, Army Military Medical University, Chongqing, 400038 PR China; 5 Department of Thoracic Surgery, Second Affiliated Hospital, Army Military Medical University, Chongqing, 400037 PR China; 6 Department of Blood Transfusion, Irradiation Biology Laboratory, Second Affiliated Hospital, Army Military Medical University, Chongqing, 400037 PR China; 7 Department of Central Laboratory, Qianjiang Hospital, Chongqing University, Chongqing, RP 409000 China

**Keywords:** Interstitial cystitis/bladder pain syndrome, Experimental autoimmune cystitis, S100A9, Autoimmune disease, Inflammation

## Abstract

**Background:**

Interstitial cystitis/bladder pain syndrome (IC/BPS) is a chronic inflammatory disease of the bladder for which no effective therapy is currently available. Understanding the pathogenesis of IC/BPS and identifying effective intervention targets are of great clinical importance for its effective treatment. Our work focuses on elucidating the key targets and underlying mechanisms of IC/BPS.

**Methods:**

We established an experimental autoimmune cystitis (EAC) mouse model and generated gene knockout mice to elucidate key mediators triggering chronic inflammatory damage in IC/BPS through using single-cell RNA sequencing, proteomic sequencing, and molecular biology experiments.

**Results:**

Our study revealed that the infiltration and activation of macrophages, T cells, and mast cells exacerbated inflammatory bladder damage in both IC/BPS and EAC mice. Notably, cell-cell communication among bladder immune cells was significantly enhanced in EAC mice. Macrophages, as the main cell types altered in EAC mice, received and transmitted the most intensity signalling. Mechanistically, macrophages synthesized and secreted S100A9, which in turn facilitated macrophage polarization and promoted the production of pro-inflammatory cytokines. S100A9 emerged as an important pro-inflammatory and pathogenic molecule in IC/BPS and EAC. Further analysis demonstrated that S100A9 activation enhanced the inflammatory response and exacerbated bladder tissue damage in IC/BPS patients and EAC mice via TLR4/NF-κB and TLR4/p38 signalling pathways. Importantly, inhibition of S100A9 with paquinimod, as well as genetic knockout of S100A9, significantly attenuated the pathological process.

**Conclusions:**

S100A9 is an important pro-inflammatory and pathogenic molecule in IC/BPS and EAC. Targeting S100A9-initiated signalling pathways may offer a novel therapeutic strategy for IC/BPS.

**Supplementary Information:**

The online version contains supplementary material available at 10.1186/s40364-025-00763-5.

## Introduction

Interstitial cystitis/bladder pain syndrome (IC/BPS) is a chronic inflammatory disease with unknown aetiology, characterized by frequent and urgent urination, as well as suprapubic or pelvic pain upon bladder filling [1, 2]. IC/BPS predominantly affects women, with an incidence rate ranging from 0.45 to 6.5%, and also exhibits a yearly increasing rate [3, 4]. The quality of life of individuals with IC/BPS is significantly compromised. Patients frequently experience anxiety, depression, sexual dysfunction, sleep disturbances and an elevated risk of suicide. Suicide rate shows four times higher than of healthy individuals [5]. The aetiology of IC/BPS is complex and involves factors such as infection, autoimmune abnormalities, mast cell activation, and defects in the urinary tract epithelial barrier [5–7]. The diagnosis and treatment of IC/BPS remain challenging due to its unclear aetiology and complex pathophysiology [5–7]. Inadequate management of IC/BPS can lead to bladder contracture, hydronephrosis, and deterioration of renal function [8–10]. In severely affected patients, cystectomy and urinary diversion become necessary [8–10]. Therefore, a deeper understanding of the biology and aetiology of IC/BPS is crucial in clinical practice, which will help to identify appropriate targets for effective intervention and treatment.

Currently, the European Society for Study of Interstitial Cystitis (ESSIC) classifies IC/BPS into two subtypes: IC/BPS with Hunner’s lesions (ESSIC BPS type 3) and IC/BPS without Hunner’s lesions (ESSIC BPS types 1 and 2) [11]. Although ESSIC BPS type 3 is not classified as an autoimmune disease, emerging research suggests that an abnormal immune-inflammatory response contributes to its pathogenesis [12–17]. IC/BPS is strongly associated with autoimmune diseases, including Hashimoto’s thyroiditis, ankylosing spondylitis, rheumatoid arthritis, and Sjögren’s syndrome [12, 13]. Additionally, complement components and autoantibodies against nuclear and bladder epithelial antigens are activated in IC/BPS patients [12, 13]. Furthermore, pathological analysis, transcriptome, and single-cell sequencing of bladder tissue samples from IC/BPS patients have confirmed an increased infiltration of inflammatory cells (lymphocytes, macrophages, and mast cells) and an overexpression of cytokines, apoptosis-related proteins, and other inflammation-associated proteins [14–17]. These findings suggest that high immune-inflammatory responses and regulatory abnormalities drive persistence and progression of IC/BPS [12–17]. Thus, investigating the key targets and pathogenic mechanisms responsible for the abnormal immune-inflammatory response in IC/BPS is vital for developing effective therapeutic interventions.

S100A9 is an important member of the calcium-binding S100 protein family and is primarily expressed in neutrophils, monocytes, and macrophages [18–20]. Extracellularly, S100A9 plays a crucial role in promoting inflammatory cell infiltration, facilitating the release of inflammatory factors, promoting macrophage polarization, and inducing cell death. Its extracellular functions are intricately linked to Toll-like receptor 4 (TLR4) and the receptor for advanced glycation end products (RAGE) [18–20]. Recent research suggests that S100A9 is a crucial pro-inflammatory pathogenic molecule that is extensively expressed in several autoimmune diseases, including systemic lupus erythematosus, rheumatoid arthritis, and Sjögren’s syndrome [18–22]. In animal models of disease, inhibition of S100A9 expression and activation has been shown to reduce the inflammatory response and tissue damage [18–22]. The expression level of S100A9 serves as a potential biomarker for disease progression, activity, and relapse [18–22]. These findings indicate that S100A9 contributes to immunological inflammatory disorders [18–22]. Previous transcriptome sequencing and single-cell sequencing demonstrated that S100A9 is highly overexpressed in IC/BPS, but these studies did not elucidate the source or functional role [15–17]. In this study, we employed clinical specimen analysis, gene knockout (KO) mouse models, proteomic sequencing, single-cell sequencing, and molecular biological experiments to investigate the expression and role of S100A9 in IC/BPS.

## Methods

### Samples

The normal bladder tissue of six patients with bladder cancer and the paraffinized tissue of six patients with Hunner’s lesions (ESSIC BPS type 3) were collected for this study [11]. The clinical specimens were obtained from the Department of Urology, Second Affiliated Hospital, Army Medical University and Affiliated Hospital of Guangdong Medical University. This study was approved by the Ethics Committee of the Second Affiliated Hospital of the Army Military Medical University and was conducted in accordance with the Declaration of Helsinki. All study participants and their family members provided explicit consent and signed an informed consent form. The sequencing data of IC/BPS patients were obtained from the GEO database under the reference code GSE11783. Immune-related gene (IRG) data were obtained from the ImmPort database for analysis. Data processing was performed using the R software platform, and the specific analytical method used was described in our previous report [23–26].

### In vivo experiment

Female C57BL/6 mice (8–10 weeks old) were purchased from the Army Medical University Animal Centre. S100A9 whole-gene KO mice were procured from Jiangsu Jicui Yaokang Biotechnology Co., Ltd. (https://www.gempharmatech.com/) for experimental use. The mouse strain under investigation was C57BL/6JGpt-S100A9em5Cd9336/Gpt. This study was approved by the Research Council and Animal Care and Use Committee of the Army Military Medical University, China (Approval No.: AMUWEC2019414) and was conducted in accordance with animal welfare guidelines and the Declaration of Helsinki. EAC in C57BL/6 mice and the S100A9-KO cystitis animal model were established as previously reported [25–28]. In short, bladder tissue from normal mice was homogenized and digested to extract the supernatant. PBS is diluted to adjust the protein concentration to 10 mg/mL. A vaccine emulsion was then prepared by mixing 100µL of the above supernatant (or PBS alone as a control) and an equal volume of complete Freund’s adjuvant (CFA; Sigma-Aldrich, St. Louis, MO, USA) or incomplete Freund’s adjuvant (IFA; Sigma-Aldrich, St. Louis, MO, USA). 200µL of the vaccine emulsion was firstly injected subcutaneously on the back of mice at 2-week intervals to induce immunity. Paquinimod is a novel oral small-molecule drug belonging to the quinoline-3-carboxamide derivatives. It specifically targets S100A9 protein, disrupting its interaction with pro-inflammatory receptors, which can improve the progression of various autoimmune and inflammatory diseases [29–31]. Based on these findings, in this study, we used paquinimod (HY-100442, MCE, USA) to intervene in EAC mice and evaluate its therapeutic potential in IC/BPS and EAC mice cystitis. In the paquinimod intervention study, the mice were divided into control, EAC and EAC + paquinimod groups. As previously noted, mice were administered an equal volume of PBS with paquinimod (5 mg/kg body weight/day) for one week during the third week [29–31]. The S100A9-KO intervention group animals were divided into the WT group, the S100A9^−/−^ group, the WT + EAC group, and the S100A9^−/−^+EAC group. Four weeks later, mouse bladder tissue samples were collected for further investigation.

### Tandem mass tag-labelled quantitative proteomic analysis

Tandem mass tag (TMT)-labelled quantitative proteomic analysis was performed on control (*n* = 3) and EAC (*n* = 3) bladder tissues [32, 33]. The sequencing experiment was conducted with the assistance of Hangzhou Jingjie Biotechnology Co., Ltd. (http://www.ptm-biolab.com.cn/). The sequencing data analysis was consistent with our previous report [23–25].

### Single-cell RNA sequencing data analysis

Single cells were extracted from the bladder tissue of female EAC (*n* = 3) and healthy (*n* = 3) mice. We followed our established protocols for bladder tissue preparation, handling, and enzymatic isolation [23–25]. An Illumina HiSeq instrument was used for sequencing. Raw data processing was performed according to the manufacturer’s instructions, and gene expression levels were quantified as transcripts per million (TPM). Three key parameters (number of RNA features greater than 200 and less than 7500, percentage of mitochondria less than 10, and percentage of red cells less than 10) were used for filtering the raw cells. For cell clustering, Seurat was used. The data processing procedures were conducted using the R platform. All the data analysis methods used were similar to those used in our previous reports [23–25].

### Haematoxylin and eosin staining and immunohistochemistry

As we previously described, paraffin-embedded sections from IC/BPS (*n* = 6) and mouse bladder (*n* = 6) were subjected to haematoxylin and eosin (HE) staining, mast cell, and immunohistochemical staining [24–26]. Bladder sections stained with HE were objectively evaluated for histological scoring by pathologists who were blinded to the details of the investigation. Briefly, the histological slides were evaluated using a 6-point grading method (Supplementary Table 1), as documented in prior studies [34–36]. The antibodies used in this investigation were as follows: rabbit monoclonal anti-S100A9 antibody (1:400, 73425, CST, USA), mouse monoclonal anti-CD4 antibody (1:100, Thermo Fisher, USA), mouse monoclonal anti-68 antibody (1:100, sc-20060, Santa Cruz Biotechnology, USA), rabbit anti-F4/80 antibody (1:100, ab300421, abcam, USA), rabbit polyclonal anti-S100A9 antibody (1:600, 26992-1-AP, Proteintech, China) and Universal Reagent Kit mouse mouse/rabbit polymer detection system (PV-6000, Zhongshan Inc, China). As previously reported, immunohistochemical analysis was performed using average optical density values calculated with ImageJ software [25, 26].

### TUNEL staining

TUNEL staining was performed as previously described [26, 34]. TUNEL cell apoptosis detection was performed according to the manufacturer’s protocol. Analysis of TUNEL staining and determination of apoptosis index was performed using ImageJ software.

### Cystometry

Cystometry and selection of recording parameters were performed as previously described [26, 34, 36]. Mice were anaesthetised with 20% urethane (Sigma-Aldrich, St. Louis, MO, USA; product code U2500). A PE-10 catheter was inserted into the bladder to facilitate saline infusion at a rate of 1 mL/h, maintained at physiological body temperature (37–38 °C). Intravesical pressure was continuously monitored throughout the experiment. After a 30-minute stabilisation period, urodynamic parameters were recorded, including maximum bladder pressure (MBP: the maximum value of the peak micturition), micturition frequency (MF: the number of micturitions per unit time) and inter-contraction interval (ICI: the time interval between two micturitions).

### Double-label Immunofluorescence analysis

IC/BPS sections (*n* = 6) and mouse bladder sections (*n* = 6) were subjected to double-label immunofluorescence analysis. The steps of the double-label immunofluorescence procedure were performed as described in our previous reports [34, 36]. The antibodies used in this investigation were as follows: rabbit monoclonal anti-S100A9 antibody (1:400, 73425, CST, USA), mouse monoclonal anti-68 antibody (1:100, sc-20060, Santa Cruz Biotechnology, USA), rabbit anti-F4/80 antibody (1:100, ab300421, abcam, USA), rabbit polyclonal anti-S100A9 antibody (1:600, 26992-1-AP, Proteintech, China), Alexa-Fluor-488-conjugated goat anti-mouse lgG (1:1000, A28175, Thermo Fisher, USA), and Alexa-Fluor-555-conjugated donkey anti-rabbit lgG (1:400, A-31572, Thermo Fisher, USA). The average fluorescence intensity of S100A9 was analysed using Image J software.

### Western blotting analysis

The steps of the Western blotting procedure were performed as described previously [26, 33–39]. Fifty micrograms of protein was subjected to electrophoresis. The antibodies used in this study were as follows: rabbit monoclonal anti-S100A9 antibody (1:800, 73425, CST, USA), mouse monoclonal anti-TLR4 antibody (1:1000, MA5-16216, Thermo Fisher, USA), mouse monoclonal anti-MyD88 antibody (1:1000, 67969-1-Ig, Proteintech, China), rabbit polyclonal anti-P38 (1:1000, B-IO-10130, Biozellen, USA), rabbit polyclonal p-p38 (1:800, B-IO-10131, Biozellen, USA), rabbit monoclonal anti-NF-κB (1:1000, 8242, CST, USA), rabbit monoclonal anti-p-NF-κB (1:800, 3033, CST, USA), mouse monoclonal anti-IκBα antibody (1:1000, 4814, CST, USA), mouse monoclonal anti-IκBα antibody (1:1000, 4814, CST, USA), rabbit monoclonal anti-IκBα antibody (1:1000, 2859, CST, USA), mouse monoclonal anti-IL-1β (1:800, sc-12742, Santa Cruz Biotechnology, USA), mouse monoclonal anti-IL-6 (1:500, sc-57315, Santa Cruz Biotechnology, USA), TNF-α (1:1000, 17590-1-AP, Proteintech, China), rabbit polyclonal anti-TNF-α (1:600, 17590-1-AP, Proteintech, China), mouse monoclonal anti-GAPDH (1:1000, 60004-1-Ig, Proteintech, China), mouse monoclonal anti-caspase-3 (1:600, 66470-2-Ig, Proteintech, China), rabbit polyclonal anti-caspase-8 (1:600, 13423-1-AP, Proteintech, China), rabbit monoclonal anti-caspase-1 antibody (1:800, 24232, CST, USA), mouse monoclonal anti-Bax (1:1000, 60267-1-Ig, Proteintech, China), mouse monoclonal anti-UPK3A (1:800, sc-166808, Santa Cruz Biotechnology, USA), rabbit polyclonal anti-UPK2 (1:600, 21149-1-AP, Proteintech, China), goat anti-rabbit secondary antibody (1:2000, G6120, Thermo Fisher, USA) and goat anti-mouse secondary antibody (1:2000, G21040, Thermo Fisher, USA). Finally, the ECL substrate and bioanalytical imaging system were used to detect the protein bands. The protein band images were collected, and the relative optical density (R.O.D.) was analysed using ImageJ software.

### In vitro experiment

The protocol used for isolating abdominal macrophages from female C57BL/6 mice (8–10 weeks old) was in accordance with previously published methods [37, 38]. At four different time points after stimulation with LPS (1 µg/mL), the concentration of S100A9 in the peritoneal macrophage supernatant was determined via an S100A9 ELISA kit. All ELISA kits were used according to the manufacturer’s protocol, and the proteins were measured at 450 nm with an ultraviolet microplate reader. Mouse-derived peritoneal macrophages were pretreated for 24 hours with 0, 10, 50, or 100 ng/mL S100A9 (HY-P74583, MCE, USA). Total RNA was first isolated using TRIzol reagent (Invitrogen, CA, USA). cDNA was synthesised from the RNA samples using a reverse transcription kit (Takara, Dalian, China). The mRNA expression levels of IL-6, TNF-α, and IL-1β were quantitatively assessed using a SYBR Premix Ex Taq™ II kit. The primers used were as follows: IL-6, forward, 5’-GTTCTCTGGGAAATCGTGGA-3’; reverse, 5’-TGTACTCCAGGTAGCTATGG-3’; TNF-α, forward, 5’-ACCACGCTCTTCTGTCTACT-3’; reverse, 5’-AGGAGGTTGACTTTCTCCTG-3’; IL-1β, forward, 5’-ACTGTTTCTAATGCCTTCCC-3’; reverse, 5’-ATGGTTTCTTGTGACCCTGA-3’; GAPDH, forward, 5’-AGGTTGTCTCCTGCGACTTCA-3’; reverse, 5’-TGGTCCAGGGTTTCTTACTCC-3’. The mRNA expression levels were calculated using the 2-^△△Ct^ method. On the other hand, peritoneal macrophages were incubated with antibodies (1:200) at 4 °C for 30 min, followed by 3 washes in FACS buffer. The antibodies used included PerCP/Cy5.5-conjugated anti-mouse CD45 antibody (#103132; BioLegend), APC conjugated anti-mouse F4/80 antibody (#123116; BioLegend), PE/Cy7-conjugated anti-mouse CD206 (MMR) antibody (#141704; BioLegend), and Alexa Fluor700-conjugated anti-mouse CD11c antibody (#117320; BioLegend). CD45^+^F4/80^+^CD11c^+^CD206^−^ cells were designated M1-like macrophages. CD45^+^F4/80^+^ CD11c^−^CD206^+^ cells were designated M2-like macrophages. Flow cytometry data were analysed with FlowJo software version 11. Mouse peritoneal macrophages were pretreated with 10, 50, or 100 mol/mL paquinimod (HY-100442, MCE, USA) for 6 h and subsequently treated with a combination of S100A9 (100 ng/mL) for 24 h. The concentrations of IL-6, TNF-α, and IL-1β in the peritoneal macrophage supernatant were determined using IL-6 (E-EL-M0044c), TNF-α (E-EL-M3063a), and IL-1β (E-EL-M0037c) ELISA kits. The protocol followed the manufacturer’s instructions precisely, and the absorbance was measured at 450 nm with an ultraviolet microplate reader from Thermo Scientific Corporation (Massachusetts, USA). Cells were harvested and prepared as described in our previous report [37, 38], and Western blotting was performed as previously reported [26, 33–36]. The concentration of antibodies used was the same as that used for the tissue immune response analysis.

### Statistical analysis

SPSS 20.0 software was used for statistical analysis. Kolmogorov-Smirinov’s one-sample test was used to evaluate the normality of the distribution of continuous variables before performing further comparisons. Normally distributed measurement data are expressed as the mean ± standard deviation (M ± SD), and comparisons between groups were performed by t tests. The quantified data are presented as the frequency or rate (%), and comparisons were made using the chi-square test. Multigroup comparisons of means were performed using one-way ANOVA or Kruskal-Wallis test, depending on the distribution of variance, with post hoc comparisons using the Student-Newman-Keuls test. Bonferroni correction was used for adjustment. *P* < 0.05 was considered to indicate statistical significance (NS: not significant, ^*^P < 0.05, ^**^P < 0.01, ^***^P < 0.001).

## Results

### Immune-inflammatory signalling plays a key role in the development of IC/BPS and EAC

**Fig. 1:**
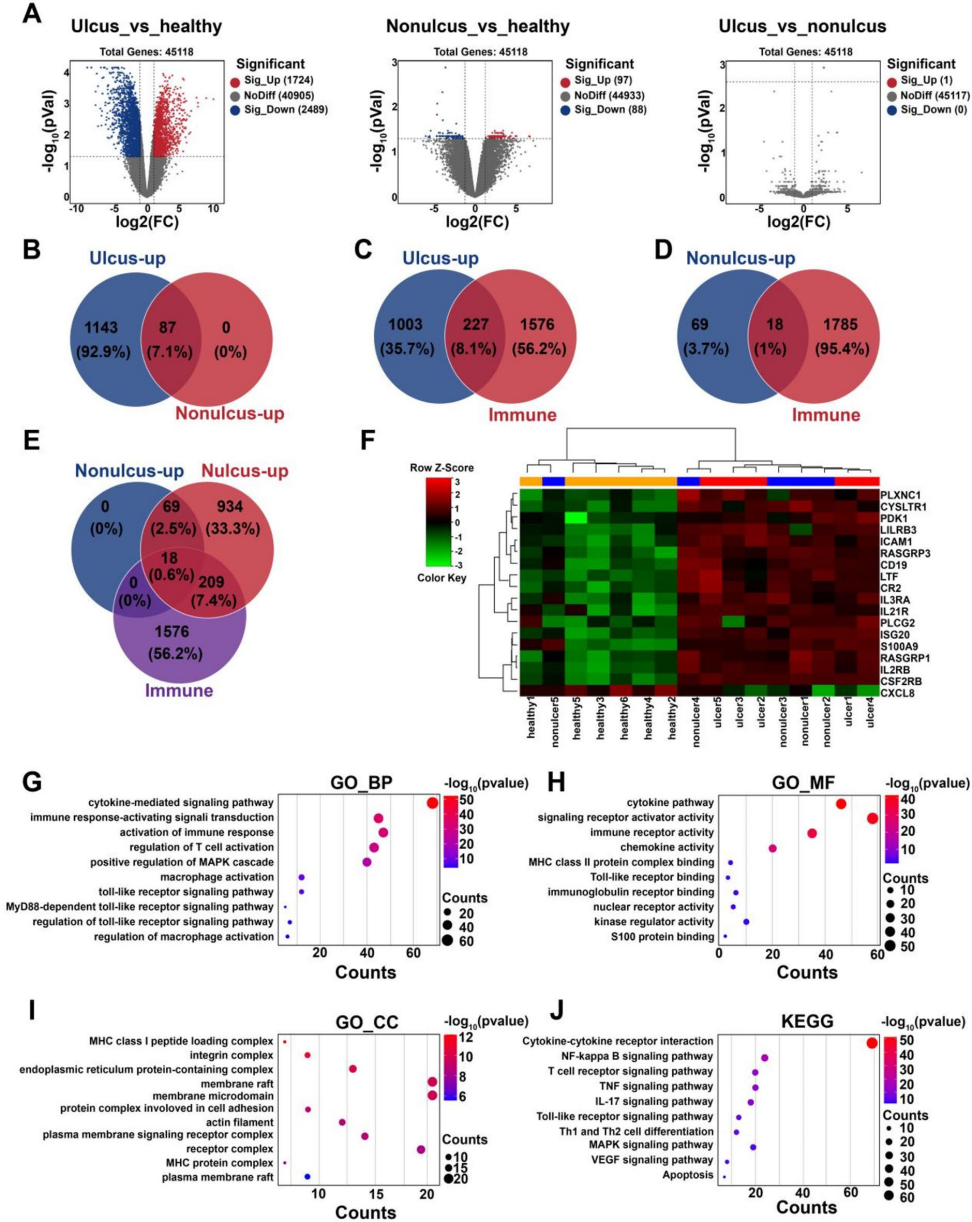
Analysis of GSE11783 data for IC/BPS patients. (**A**)Volcano plot of DEGs in the ulcer, non-ulcer, and normal groups. (**B**) Venny plot of upregulated genes in the ulcer and non-ulcer groups. (**C**-**D**) Venny plots of upregulated genes and immune-related genes in the ulcer and non-ulcer groups. (**E**) Venny plots of upregulated genes and immune-related genes in the ulcer group and non-ulcernonulcer group. (**F**) Heatmap plot of the expression of 18 overlapped genes from. (**G**-**J**) GO and KEGG enrichment analysis of 227 overlapping genes

To elucidate the mechanism behind IC/BPS, RNA-seq data from human IC/BPS bladder tissues (obtained from the public database GSE11783) and TMT proteomic sequencing data from mouse experimental autoimmune cystitis (EAC) bladder tissues were analysed. Compared to the healthy group, the ulcer group had 1724 upregulated and 2489 downregulated genes, while the non-ulcer group had 97 upregulated and 88 downregulated genes, but there were no significant differences between the ulcer and non-ulcer groups (Fig. 1A). Venny plot analysis showed that 227 ulcer upregulated genes were demonstrated immune-related genes (Fig. 1B ∼ **1D**). More importantly, 18 upregulated critical target genes in both ulcer and non-ulcer groups were found to be immune-related (Fig. 1E ∼** 1 F**). Gene Ontology (GO) and Kyoto Encyclopaedia of Genes and Genomes (KEGG) enrichment analysis of 227 ulcer upregulated immune-related genes showed immune-inflammatory signalling pathways, such as immune response activation, macrophage activation, T-cell activation, S100 protein binding, Toll-like receptors (TLRs), the TLR4/NF-κB pathway, the MAPK pathway, and apoptosis (Fig. 1G ∼** 1 J**). These results suggest that the dysregulation of immune inflammation is central to the pathophysiology of IC/BPS.

**Fig. 2:**
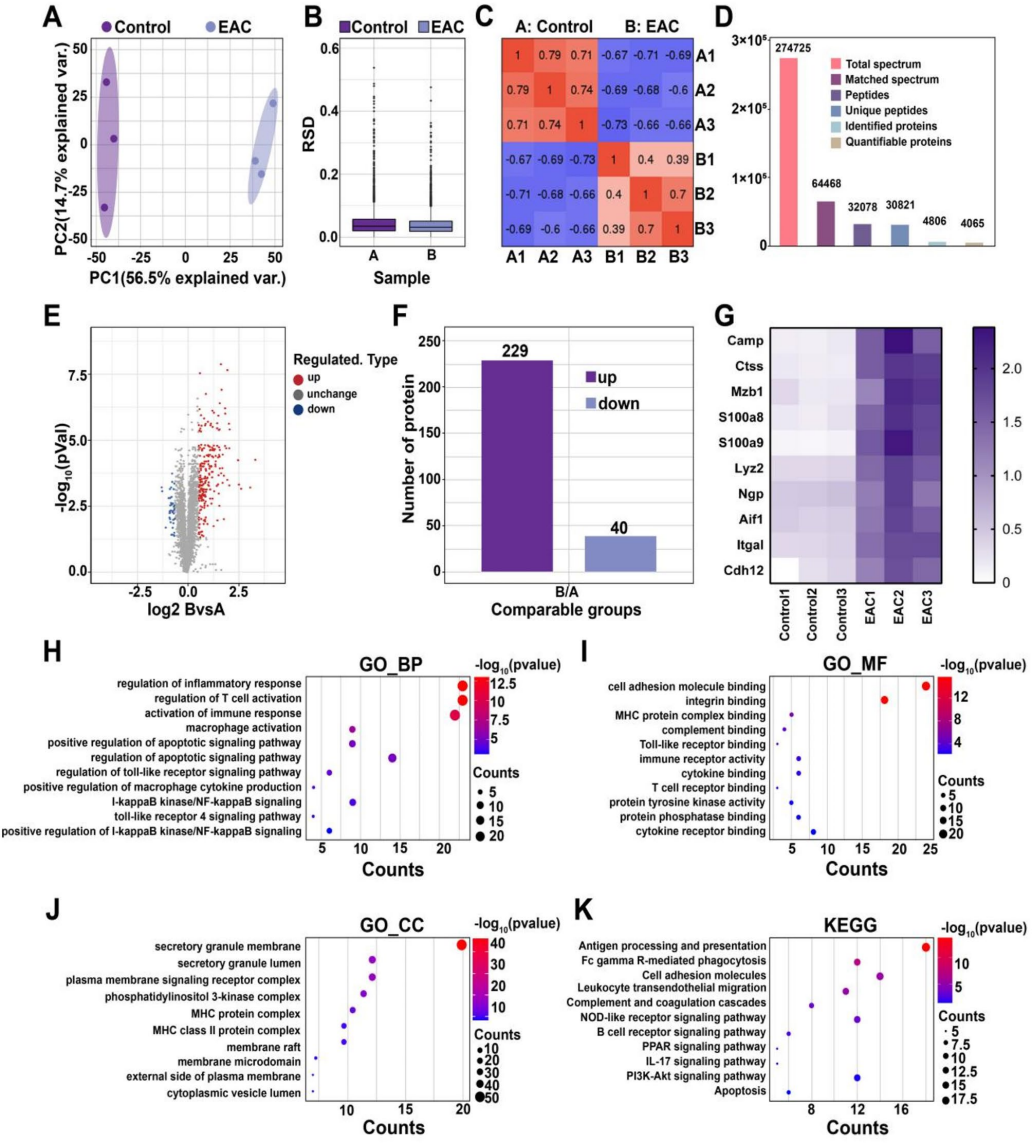
Comparison TMT analysis of proteomic sequencing between control and EAC mouse bladder tissues. (**A**) A two-dimensional scatter plot of the principal component analysis (PCA) distribution of all samples by using quantified proteins. (**B**) Box plot of the relative standard deviation (RSD) distribution of repeated samples. (**C**) Heatmap of Pearson correlation coefficients for all quantified proteins between each pair of samples (*n* = 3). (**D**) Basic statistical images of the mass spectrometry results. (**E**) Volcano plot of proteins differentially expressed between the EAC and the control group. (**F**) Column chart of the distribution of differentially expressed proteins between the EAC and control group. (**G**) Heatmap plot of the top 10 upregulated genes in the EAC group (*n* = 3). (**H**-**K**) GO and KEGG enrichment analyses of the DEGs in the EAC group

To investigate key molecules involved in the pathogenesis of IC/BPS-related inflammation, we established a mouse EAC model and performed TMT proteomic sequencing on bladder samples. As shown in Fig. 2A ∼ 2 C, the high repeatability and reliability of the sequencing analyses were validated by using principal component analysis (PCA), relative standard deviation (RSD) and Pearson’s correlation coefficient analysis. A total of 4806 proteins were identified in the current sequencing, of which 4065 were quantifiable (Fig. 2D). Protein screening was performed at a 1.5-fold expression threshold, identifying 229 upregulated and 40 downregulated proteins in the EAC mice (Fig. 2E ∼ 2 F). The top 10 ranked upregulated proteins included Camp, Ctss, Mzb1, S100a8, S100a9, Lyz2, Ngp, Aif1, Itgal, and Cdh12 (Fig. 2G). Furthermore, GO and KEGG enrichment analysis of upregulated proteins in EAC mice revealed the activation of immuno-inflammatory signalling pathway, including immune response activation, macrophage and T cell activation, TLR signalling pathway, NF-κB, the PI3K/Akt pathway and apoptosis (Fig. 2H and K). These findings were correlated with the pathologies of the IC/BPS samples mentioned above.

### S100A9 is a key immune-inflammatory target molecule for IC/BPS and EAC

**Fig. 3:**
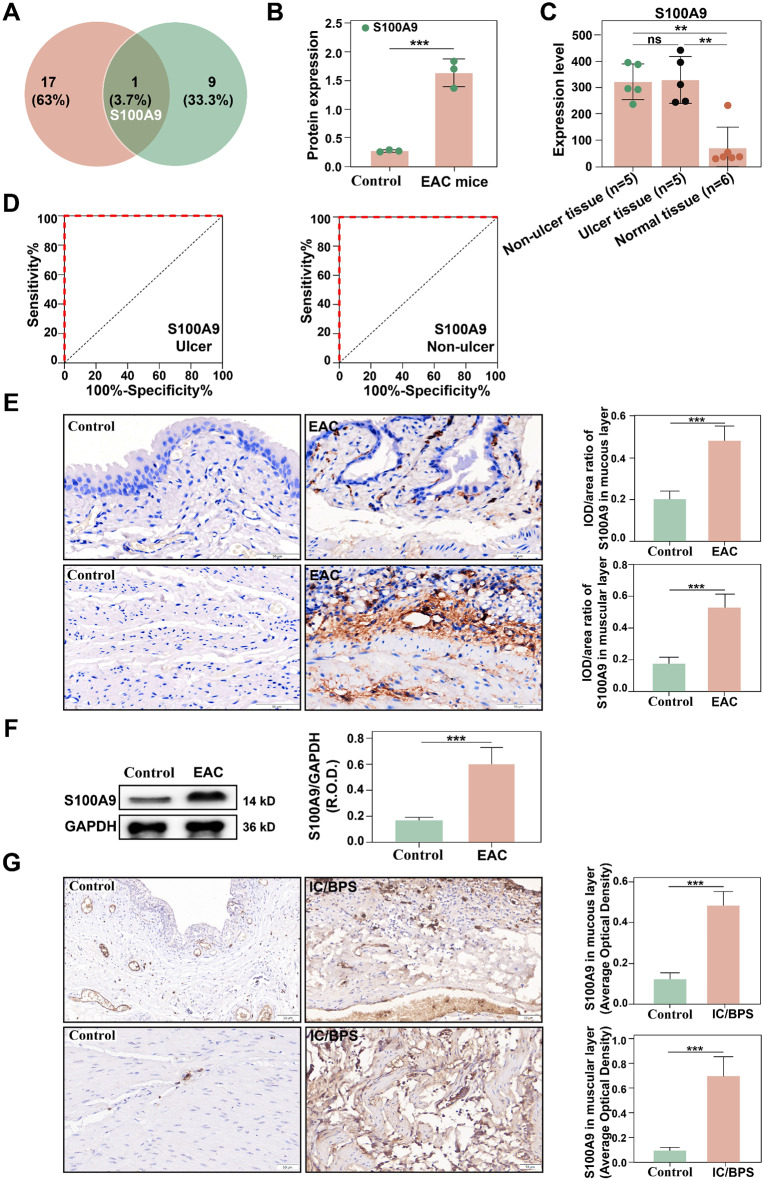
Screening and analysis of the core target molecule S100A9. (**A**) Venny plot of the top 10 genes upregulated in the EAC group and 18 candidate key genes for IC/BPS. (**B**-**C**) Analysis of S100A9 expression in the bladders from EAC mice and IC/BPS patients. (**D**) ROC curve analysis of S100A9 gene expression in the ulcer and non-ulcer groups of patients with IC/BPS. (**E**) Immunohistochemical analysis of S100A9 expression in EAC mice (×200, *n* = 6). (**F**) Western blot analysis of S100A9 expression in EAC mice. (**G**) Immunohistochemical analysis of S100A9 expression in IC/BPS patients (×200, *n* = 6). NS indicates no difference; *p < 0.05, **p < 0.01, ***p < 0.001

To identify the key molecule implicated in the pathogenesis of IC/BPS-related immune inflammation, we performed an overlapping analysis of the 18 key IC/BPS genes and the top 10 elevated genes in the EAC group. This analysis revealed that S100A9 was the most critical target gene (Fig. 3A). Further expression analysis confirmed that S100A9 was overexpressed in both IC/BPS patients and EAC mice (Fig. 3B ∼ 3 C). To establish the reliability of S100A9 gene, a receiver operating characteristic (ROC) curve analysis was performed for the ulcer and non-ulcer groups (Fig. 3D). We discovered that S100A9 expression exhibited 100% sensitivity and specificity in both groups, highlighting its pathogenic significance. By using immunohistochemistry and Western blotting (Fig. 3E ∼** 3G**), we found increased S100A9 in the bladder mucosa and muscles of EAC mice (Fig. 3E ∼** 3 F**), particularly in IC/BPS patients (Fig. 3G, *p* < 0.001). Research has indicated that S100A8 and S100A9 can assemble into homodimers or heterodimers, which perform vital physiological functions both in vitro and in vivo [18–22]. Therefore, we also verified the expression of S100A8. We found that S100A8 was highly expressed in the IC/BPS ulcer and nonulcer groups (*P* < 0.01; Supplementary Fig. 1A), also in the bladders of EAC mice (*p* < 0.001; Supplementary Fig. 1B). ROC analysis demonstrated that S100A8 overexpression exhibited high sensitivity and specificity (Supplementary Fig. 1C). Moreover, immunohistochemical analysis indicated elevated S100A8 expression levels in both EAC mouse and IC/BPS patient samples (*p* < 0.001; Supplementary Fig. 1D ∼ 1E). These findings indicate that both S100A8 and S100A9 play a role in IC/BPS and EAC pathogenesis. However, previous research has suggested that targeted deletion of S100A9 in mice ( S100A9 KO) results in a loss of S100A8 and S100A9, probably because of greater turnover of isolated S100A8 in the absence of its binding partner S100A9 [18–22, 29–31]. As a result, we selected S100A9 as the focal molecule to gain a better understanding of the role of S100A8 and S100A9 in bladder function.

### Neutrophil and macrophage-derived S100A9 involved in the inflammatory response of IC and EAC

**Fig. 4:**
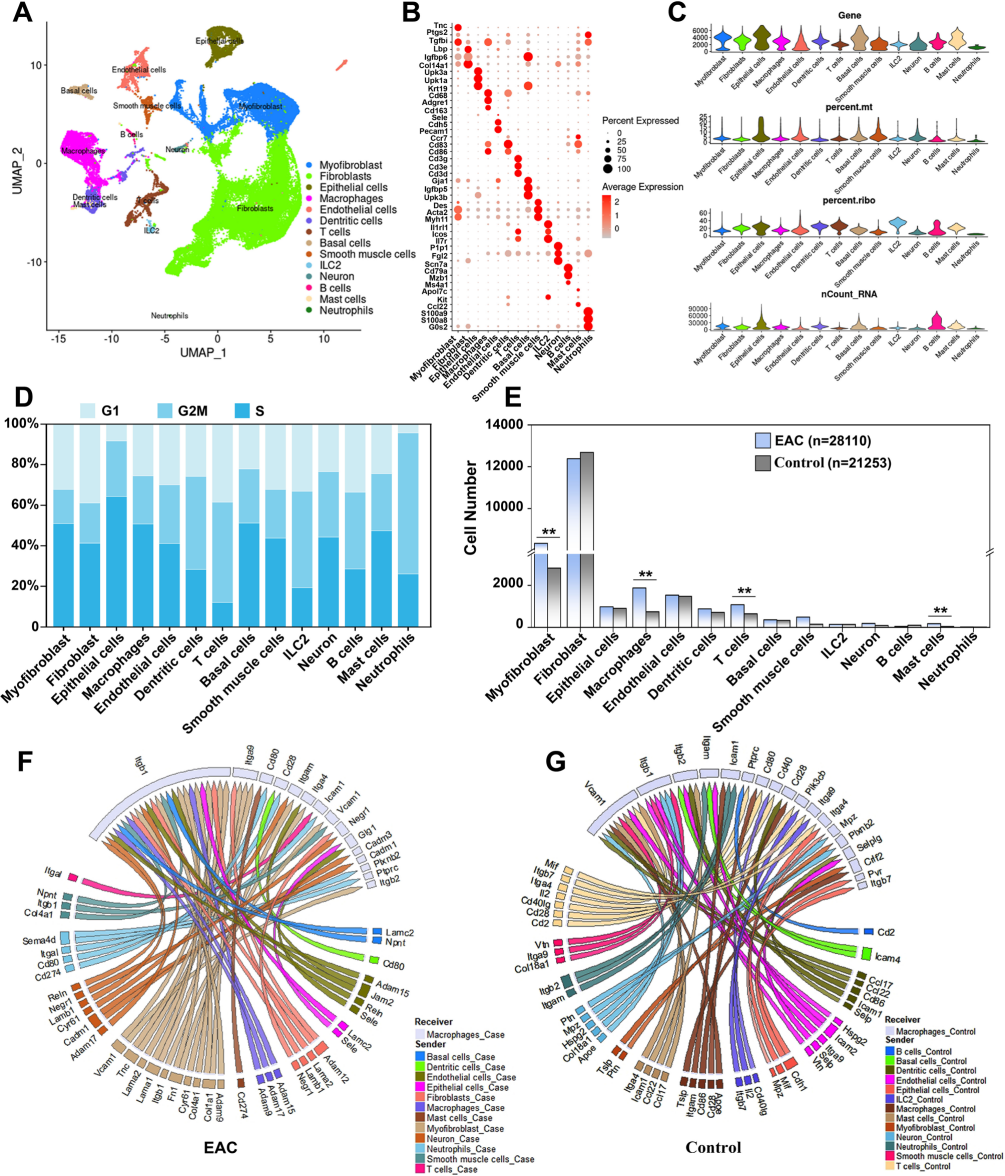
Analysis of single cell types in the control and EAC groups. (**A**) UMAP cluster of whole bladder cells in mice. (**B**) Dot plots showing the expression of three key markers in each cell types. (**C**) Distribution of the gene expression, mitochondrial ratio, ribosomal ratio, and total RNA count in different cell types. (**D**) Comparison of cell cycle phases between different cell types. (**E**) Analysis of cell number in different cell types between EAC and control group. (**F**) Circle plot showing the key signals received by macrophages in the EAC group. (**G**) Circle plot showing the key signals received by macrophages in the control group

To investigate the mechanism of S100A9 activation in EAC mouse development, we performed single-cell sequencing of bladder tissues from EAC and healthy mice. A total of 49,363 cells from the control group (*n* = 21253) and EAC model group (*n* = 28110) were obtained for cluster analysis after strict filtering and quality control (average gene expression > 1000). Uniform manifold approximation and projection (UMAP) analysis identified 14 distinct cell types in the bladder tissues of both control and EAC groups, including control bladder tissue and all immune cells (Fig. 4A, Supplementary Fig. 2A). We defined each cell type with three example gene tags (Fig. 4B). Single-cell sequencing results are typical, trustworthy, and suitable for testing data extraction according to the four key quality control standards (Fig. 4C). Cell cycle analysis of all cell types revealed that immune cells were in the proliferative phase, indicating that the immune system was activated in EAC bladders (Fig. 4D, Supplementary Fig. 2B). Compared to the control group, the number of myofibroblasts (*P* < 0.01), macrophages (*P* < 0.01), T cells (*P* < 0.01), and mast cells (*P* < 0.05) in the EAC group was notably increased (Fig. 4E). To investigate differences in signalling interactions between the bladder tissue cells in EAC and control mice, we performed NichNet signalling analysis. The results showed that macrophages in the bladder tissue of the EAC group received strong Itgb1 signals (Fig. 4F, Supplementary Fig. 2C). whereas macrophages in the control group did not exhibit significant stimulatory signalling (Fig. 4G, Supplementary Fig. 2D). Additionally, the CellChat program was employed to assess cell-cell communication. The results indicated that the EAC group had significantly higher levels of communication between cells than the control group (Supplementary Fig. 2E and F, Supplementary Fig. 3A). The heatmap shows differences in both the number and intensity of interactions between different cell types in the EAC and control groups (Supplementary Fig. 3B). In the heatmap, red represents an enhanced signal in the EAC group, while blue represents a weakened signal in the EAC group (Supplementary Fig. 3B). Circular visualization further revealed that the enhanced signal (red) in the EAC group was present mainly in myofibroblasts and immune cells, while the reduced signal (blue) was distributed mainly in fibroblasts and basal cells (Supplementary Fig. 3C). Furthermore, we created a bar chart to illustrate variances in information flow throughout the network between the EAC group and the control group. Red signals were mainly present in the control group, and green signals were primarily present in the EAC group. We found that several immune response signals, including IL6, IL10, IL16, and MHC-II, exhibited high enrichment in the EAC group (Supplementary Fig. 3D). Moreover, the network map indicates that more immune signals were interconnected with myofibroblasts, macrophages, T cells, and mast cells (Supplementary Fig. 3E). Notably, we also found increased expression of TGFB and MHC-II signals in macrophages from EAC mouse bladders (Supplementary Fig. 3F). This finding was consistent with that of the NicheNet enhancement study of Itgb1 signals (Fig. 4F). Furthermore, in comparison control, macrophages in the EAC group mainly received an amplified Ptgrc-Mrc1 signal, whereas the weakened signal was primarily attributed to Ape-Cd74 (Supplementary Fig. 3G). Moreover, in EAC model mice, the upregulated signal of macrophages was CCL8, while the downregulated signal was mainly App-Cd74 (Supplementary Fig. 3H). These results suggested that the increased number of macrophages in the EAC group may mediate the regulation of the immune-inflammatory response in mice with EAC.

**Fig. 5:**
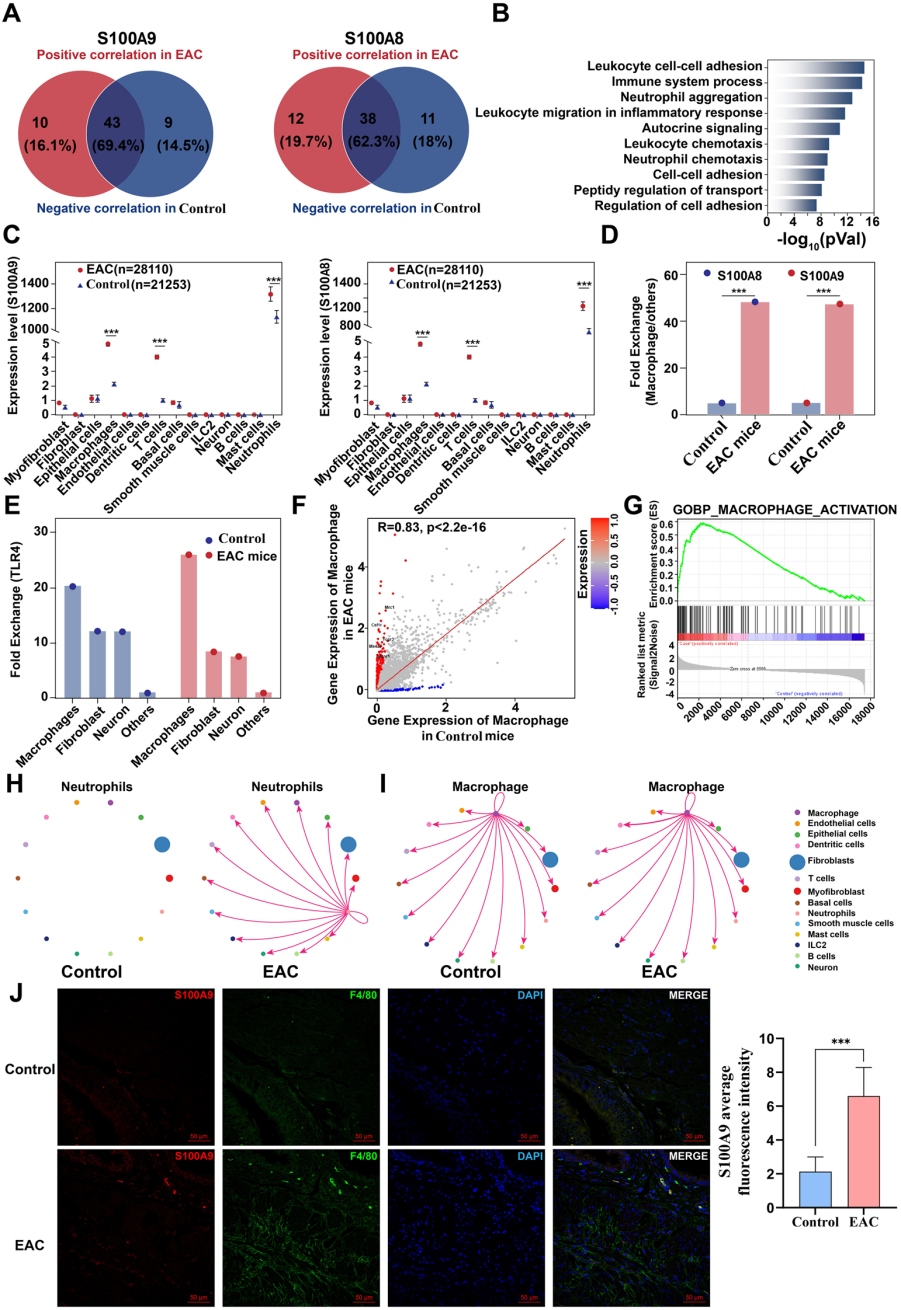
Expression analysis of S100A9 in EAC group from data of single-cell sequencing. (**A**) Venny plot of overlapping genes related to S100A9 and S100A8 comparing within the EAC and control groups. (**B**) GO enrichment analysis of gene pathways associated with S100A9 and S100A8. (**C**) Distribution and expression analysis of S100A9 and S100A8 in different bladder cell types between control and EAC group. (**D**) Comparative analysis of S100A9 and S100A8 expression in macrophages between control and EAC groups. (**E**) Expression analysis of TLR4 receptor in bladder cells between control and EAC groups. (**F**) Comparative analysis of gene expression in macrophages between the EAC and control groups. (**G**) GSEA analysis of the activation pathway of MACROPHAGE ACTIVATION. (**H**) Analysis of cell-cell communication source of neutrophils between control and EAC group. (**I**) Analysis of cell-cell communication source of macrophages between control and EAC group. (**J**) Immunofluorescence confocal analysis of S100A9 and macrophage marker F4/80 in control and EAC groups (*n* = 6)

Except for macrophage activation in EAC bladder, we analysed gene expression that were positively and negatively correlated with S100A9 and S100A8 in single-cell sequencing data from both the EAC and control group. 10 genes positively associated with S100A9 and 12 genes positively correlated with S100A8 were identified in the EAC group. (Fig. 5A). These gene signalling pathways revealed strong correlations with immune activation, inflammatory response, immune cell adhesion, infiltration, and chemotaxis (Fig. 5B). More specifically, S100A9 and S100A8 were mainly secreted by neutrophils, macrophages, and T cells (Fig. 5C). However, S100A9 and S100A8 increased significantly more in macrophages than in neutrophils in the EAC group compared to the control group (Fig. 5D, P **< 0.001**). Interestingly, compared to other cells, the downstream receptors TLR4 of S100A9 and S100A8 exhibited highest expression levels in EAC bladder macrophages (Fig. 5E). Further compared macrophage molecule expression between the EAC and control group showed a significant increase in the expression of the key molecules Mcr1, Csf1r, and Fcgr3. These results suggested that macrophages are activated in the EAC (Fig. 5F), which was supported by Gene Set Enrichment Analysis (GSEA) pathway analysis (Fig. 5G).

**Fig. 6:**
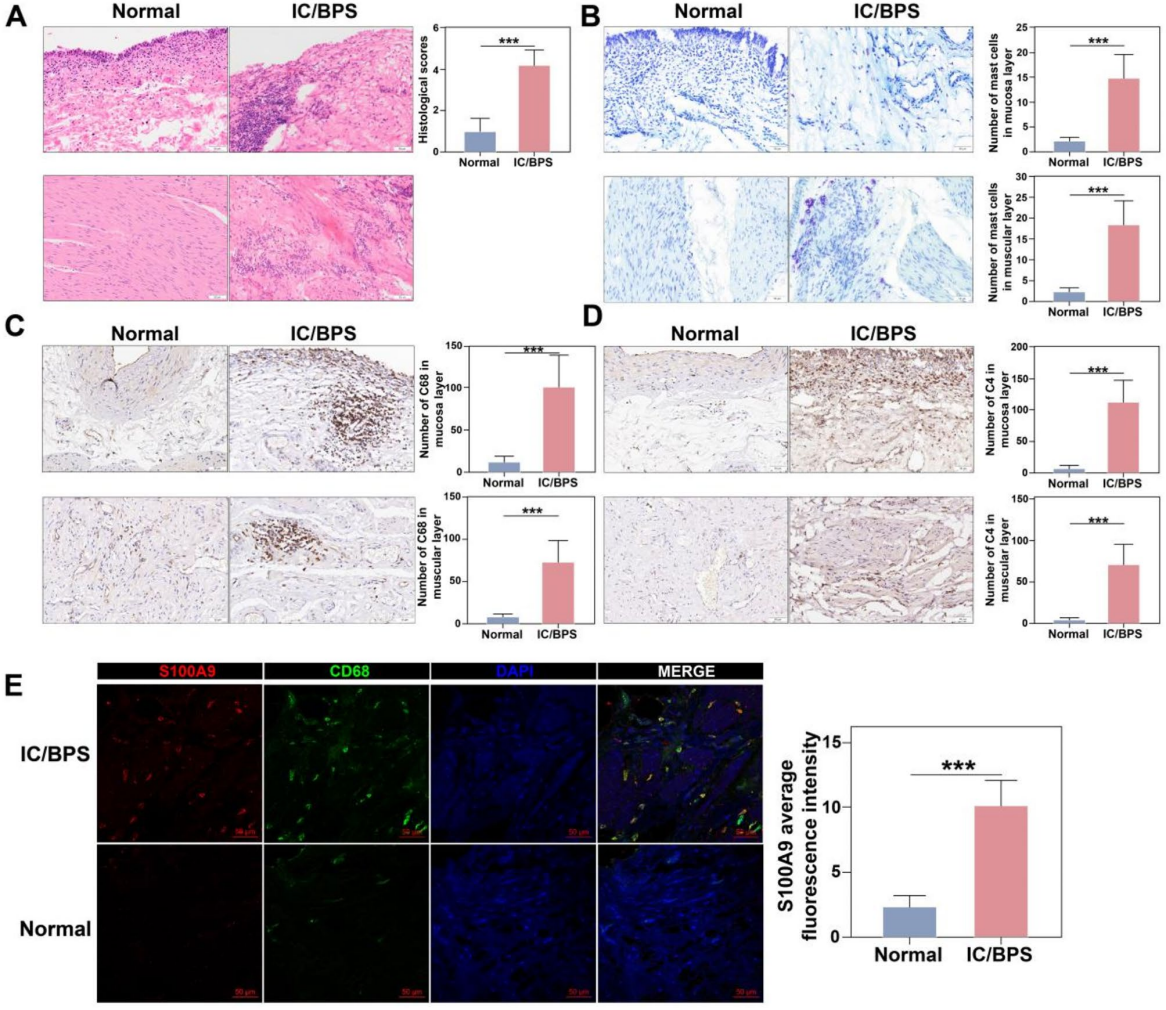
Analysis of S100A9 expression and immune cell infiltration in IC/BPS bladder specimens. Analysis of HE staining of bladder in patients with ulcer type of IC/BPS (×200, *n* = 6). (**B**) Analysis of bladder mast cell staining in patients with ulcer type of IC/BPS (×200, *n* = 6). (**C**) Analysis of CD68 staining of bladder macrophages from ulcer type IC/BPS patients (×200, *n* = 6). (**D**) CD4 staining analysis of bladder T cells from patients with ulcer type of IC/BPS (×200, *n* = 6). (**E**) Confocal immunofluorescence analysis of S100A9 and CD68 in normal and IC/BPS groups (*n* = 6). NS indicates no difference; *p < 0.05, **p < 0.01, ***p < 0.001

Since the key receptor of S100A8/S100A9 is expressed mainly in macrophages (Fig. 5E), we performed a separate study of neutrophil-macrophage communication. Although there was no intercellular communication from neutrophils in the control group, there was increased signalling from neutrophils in the EAC group (Fig. 5H). Interestingly, we found intercellular communication between macrophages and neutrophils in EAC (Fig. 5I). Subsequently, immunofluorescence confirmed the colocalization of S100A9 and macrophages, as well as a significant increase in S100A9 expression in the EAC group (Fig. 5J). These results suggest that S100A8 and S100A9 secreted by neutrophils may act on macrophages to exert their biological effects. Moreover, GO, KEGG and GSEA analyses of highly expressed genes identified through single-cell sequencing in the EAC group revealed that various signalling pathways were activated, including the immune-inflammatory response, macrophage activation, T-cell activation, Toll-like receptor signalling, MAPK, PI3K/Akt, TNF, and apoptosis (Supplementary Fig. 4A-E). To validate S100A9/Macrophages signalling in clinical IC/BPS patients, we collect bladder tissues samples from IC/BPS and unaffected patients. Higher histological scores were present in IC/BPS bladder tissues (Fig. 6A). Additionally, significant increases in mast cells (Fig. 6B), macrophages (Fig. 6C), and T cells (Fig. 6D) were observed both in mucosa and in muscular of bladder in IC/BPS patients. Immunofluorescence confirmed the colocalization of increasing S100A9 and macrophages in the IC/BPS group (Fig. 6E). These changes were consistent with IC/BPS data and EAC TMT proteomics data, confirming that activation of the immune inflammatory response is involved in the pathophysiology of EAC and IC/BPS. Based on our previous research findings, we hypothesise that neutrophil-derived S100A9 and S100A8 activate macrophages through the TLR4 signalling pathway, leading to immune-inflammatory injury in IC/BPS and EAC bladder.

### S100A9 activates macrophage polarisation and promotes the release of inflammatory factors

**Fig. 7:**
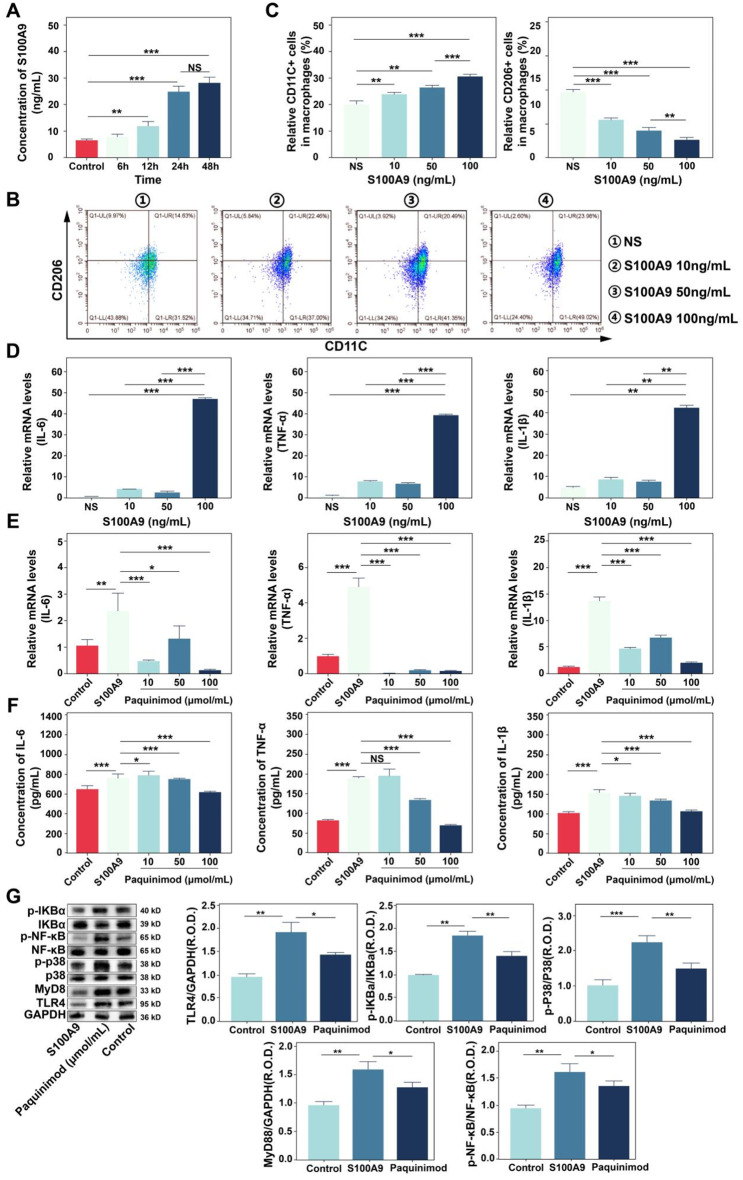
S100A9 promotes macrophage activation and secretion of inflammatory factors. (**A**) LPS promoted S100A9 secretion in mice primary macrophages (*n* = 6). (**B**-**C**) S100A9 promoted pro-inflammatory polarisation of primary mouse macrophages (*n* = 3). (**D**) S100A9 promoted IL-6, TNF-α, and IL-1β expression in mouse primary macrophages (*n* = 6). (**E**) Paquinimod inhibited the increased IL-6, TNF-α and IL-1β mRNA expression in S100A9-induced mouse macrophages (*n* = 9). (**F**) Paquinimod inhibited the increase of IL-6, TNF-α and IL-1β levels in S100A9-induced mouse macrophages (*n* = 9). (**G**) S100A9 activated TLR4/NF-κB and TLR4/p38 signalling pathways in primary mouse macrophages (*n* = 3). NS indicates no difference; *p < 0.05, **p < 0.01, ***p < 0.001

To further elucidate the causality relationship between macrophages and S100A9, mice primary macrophages were employed for validation. The results indicated that the expression of S100A9 increased over time after LPS stimulation of primary mouse macrophages, reaching a peak level at 48 h (Fig. 7A). Furthermore, Stimulated mouse primary macrophages with active S100A9 protein, M1-polarised macrophages gradually increased (Fig. 7B ∼** 7 C**), but M2-polarised macrophages decreased as the S100A9 concentration rising (Fig. 7B ∼** 7 C**), which indicated that S100A9 regulates macrophage pro-inflammatory activation. In addition, the mRNA expression levels of the IL-1β, IL-6 and TNF-α significantly increased in macrophages, peaking at a concentration of 100 ng/mL (Fig. 7D). Previous studies have reported that S100A9 contributes to the onset and progression of immune-inflammatory disorders by activating the TLR4/NF-κB and TLR4/P38 signalling pathways [18–22, 31, 40]. Therefore, the S100A9-blocking drug paquinimod was used to investigate whether it contributes to the release of macrophage inflammatory factor via the TLR4/NF-κB or TLR4/P38 signalling pathways. The S100A9-specific blocker paquinimod has been shown to have anti-inflammatory and antiapoptotic properties and alleviate tissue damage by preventing S100A9 from binding to TLR4 [18–22]. As shown in Fig. 7E, treatment with paquinimod (10, 50, or 100 µmol/mL) significantly reduced the mRNA expression of IL-1β, IL-6, and TNF-α in macrophages stimulated with S100A9 (100 ng/mL) with a dose-dependent way. TNF-α, IL-6, and IL-1β expression decreased in a dose-dependent way in the supernatant of macrophage culture medium by using Enzyme-linked immunosorbent assay (ELISA) technology (Fig. 7F). Western blot analysis demonstrated that S100A9 can activate proteins of the TLR4/NF-κB and TLR4/P38 signalling pathways in S100A9-stimulated primary macrophages, but paquinimod was found to significantly inhibit the performance in the concentration of 50 µmol/mL (Fig. 7G). Therefore, current investigation revealed that macrophages can receive and release S100A9, which can self-activate to release inflammatory factors through the TLR4/NF-κB and TLR4/P38 signalling pathways.

### S100A9 KO and paquinimod inhibition can significantly reduce inflammatory bladder damage and immune cell infiltration in EAC mice

**Fig. 8:**
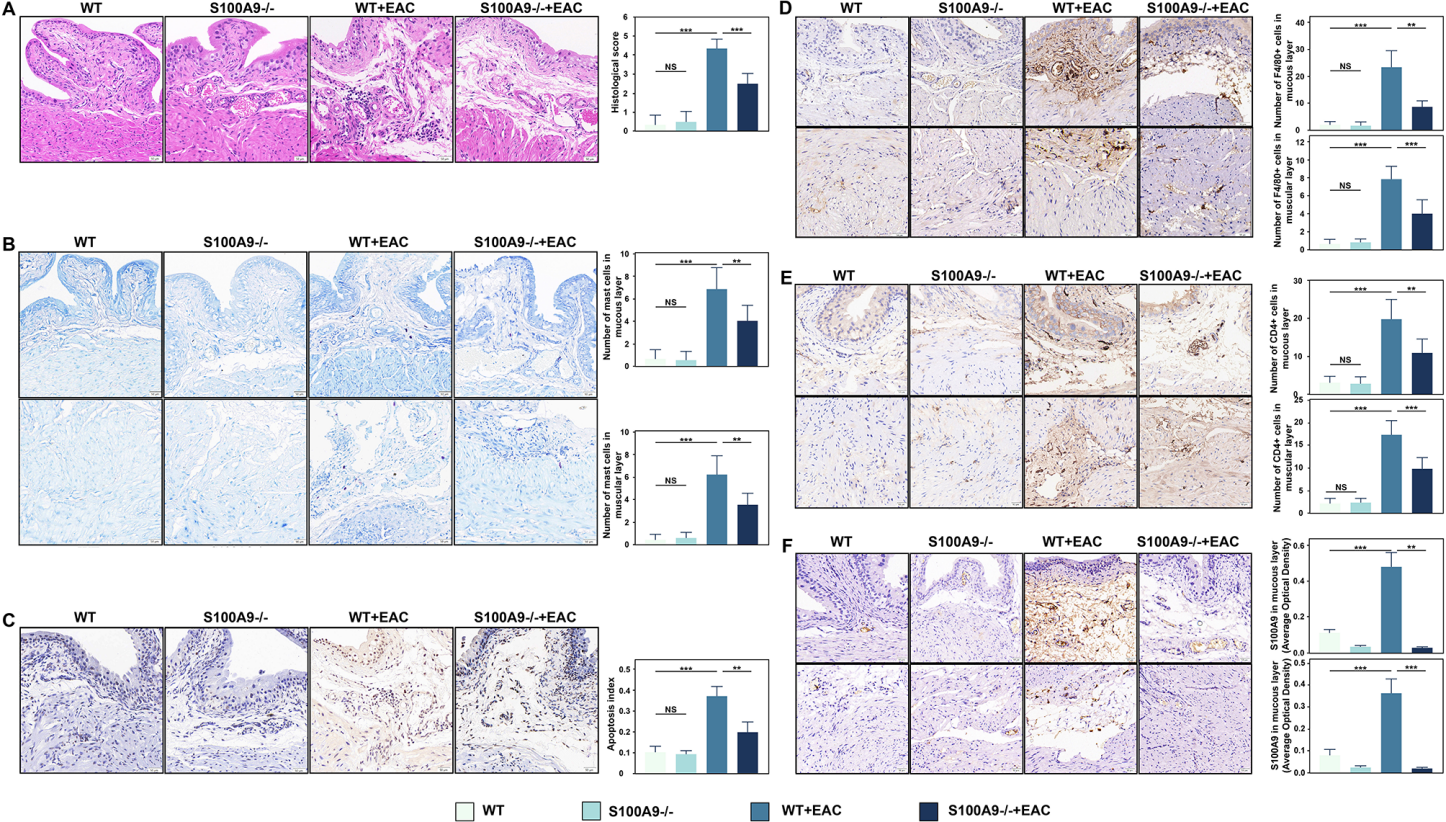
Analysis of the effects of S100A9 knockdown on bladder tissue and immune cells in EAC mice (*n* = 6). **(A)** HE staining analysis of bladder tissues in WT and S100A9 knockdout mice of control and EAC groups (×200). (**B**) Analysis of mast cell infiltration in WT and S100A9 knockout bladder tissues from control and EAC groups (×200). (**C**) TUNEL staining for apoptosis analysis in bladder tissues from WT and S100A9 knockout mice in the control and EAC groups (×200). (**D**) Immunohistochemical analysis of macrophage marker F4/80 in WT and S100A9 knockout mice of control and EAC groups (×200). (**E**) Immunohistochemical analysis of CD4 in WT and S100A9 knockout mice of control and EAC groups (×200). (**F**) Immunohistochemical analysis of S100A9 in WT and S100A9 knockdout mice of the control and EAC groups (×200). NS indicates no difference; *p < 0.05, **p < 0.01, ***p < 0.001

**Fig. 9:**
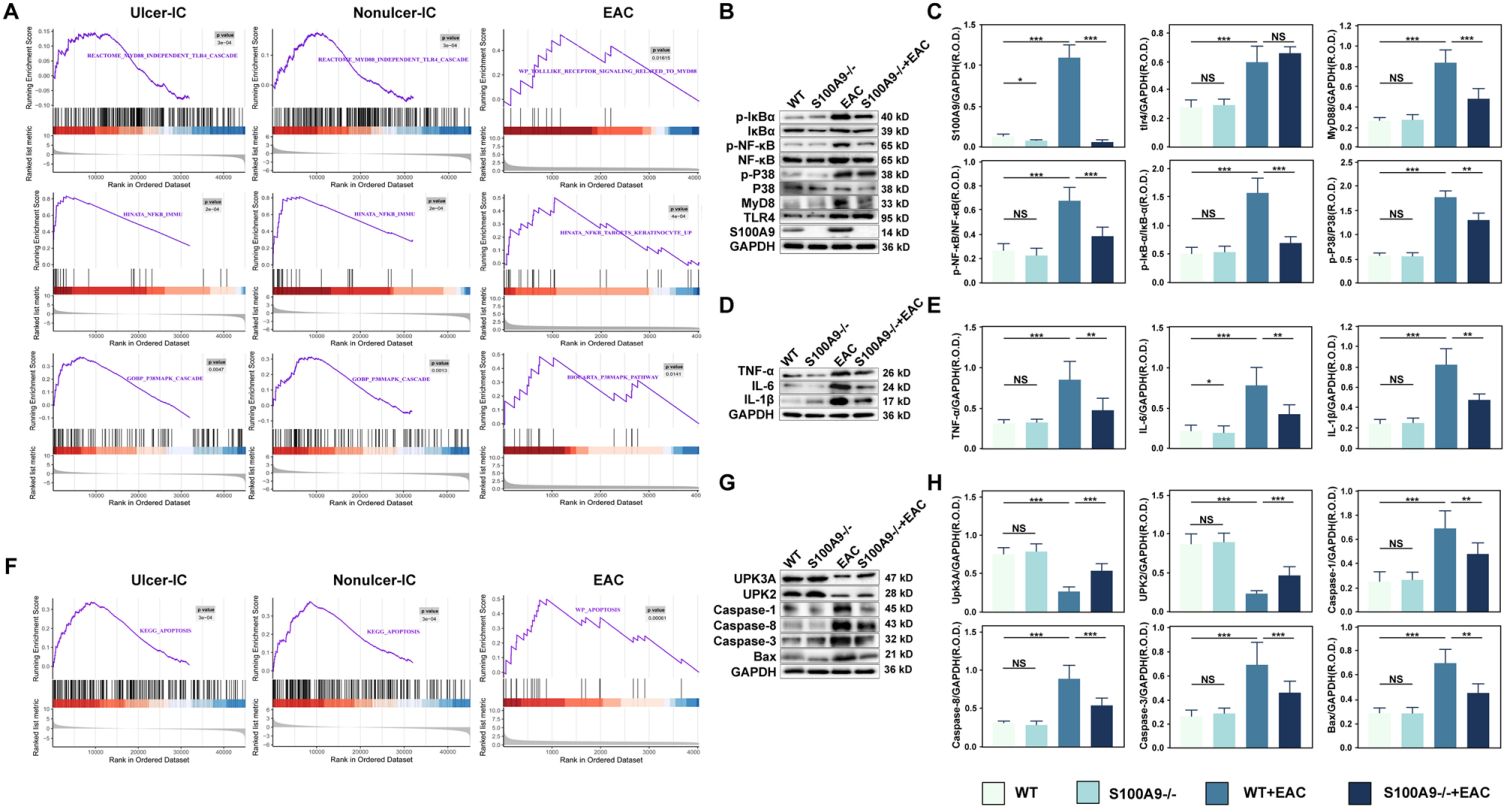
Knockdown of S100A9 significantly reduced TLR4/NF-κB and TLR4/p38 signalling pathway activating and decreasing inflammation and apoptosis-related protein expression in EAC mice (*n* = 6). GSEA analysis showing that TLR4/MyD88, NF-κB, and p38 signalling are activated in IC/BPS patients and EAC mice. (**B**-**C**) Knockdown of S100A9 significantly reduced TLR4/NF-κB and TLR4/p38 signalling pathway protein expression in EAC mice. (**D**-**E**) Knockdown of S100A9 significantly reduced the expression of bladder inflammation-related proteins (IL-6, IL-1β and TNF-α) in EAC mice. (**F**) GSEA analysis showing that apoptosis signalling is activated in IC/BPS patients and EAC mice. (**G**-**H**) Knockdown of S100A9 expression significantly reduced the expression of bladder apoptosis-related proteins (Bax, caspase-3, caspase-8, and caspase-1) and improved the expression of epithelial damage marker proteins (UPK3A and UPK2) in EAC mice. NS indicates no difference; *p < 0.05, **p < 0.01, ***p < 0.001

In S100A9^−/−^ EAC mice, paquinimod inhibition significantly reduced bladder histological scores in the EAC group compared to wild-type or healthy mice (Fig. 8A, Supplementary Fig. 5A, **p** < 0.05). Additionally, infiltrating mast cells (Fig. 8B, Supplementary Fig. 5B,** p** < 0.05), apoptosis indices (Fig. 8C, Supplementary Fig. 5C,** p** < 0.05), macrophage marker F4/80^+^ (Fig. 8D, Supplementary Fig. 5D,** p** < 0.05) and infiltrating T cells (Fig. 8E, Supplementary Fig. 5E,** p** < 0.05) were increased in the EAC group, but both S100A9^−/−^ deletion and paquinimod inhibition can rescue the process. Remarkably, S100A9 expression in the EAC bladder was considerably higher (*p* < 0.001) than in the healthy, wild-type (WT), and S100A9^−/−^ groups (Fig. 8F, Supplementary Fig. 5F). These results confirm that S100A9 deletion and paquinimod inhibition can strongly alleviate the inflammatory damage and immune cell infiltration in the bladder tissue of EAC mice. Moreover, S100A9 may promote the release of its protein and contribute to the amplification of the inflammatory cascade response in EAC mice. Bioinformatic analysis showed that inflammatory pathways such as TLR-MyD88, NF-κB and MAPK pathways were activated in both IC/BPS patients and EAC mice, which was validated by in vitro cell experiments. GSEA analysis revealed the activation of TLR4/MyD88, NF-κB, and p38 signalling in the bladders of both IC/BPS patients and EAC mice (Fig. 9A). In the EAC group, the protein expression of S100A9, MyD88, TLR4, p-NF-κB, p-IκB, and p-p38 was significantly higher than that in the control, WT, and S100A9^−/−^ group (Fig. 9B ∼** 9 C**, *p* < 0.001), but significantly decreased following knock out of S100A9. Similarly, treatment with paquinimod to inhibit S100A9, the protein expression levels of MyD88, p-NF-κB, p-IκB, p-p38, and S100A9 were significantly reduced in EAC mice (Supplementary Fig. 6A ∼ 6B, *p* < 0.05). These results indicate that both paquinimod inhibition and S100A9 knockout dramatically reduced the activation of the TLR4/NF-κB and TLR4/p38 pathways in EAC mice.

Additionally, GSEA pathway analysis demonstrated the activation of apoptotic signalling in the bladders of IC/BPS patients and EAC mice (Fig. 9F). The expression of inflammatory factors (TNF-α, IL-6, and IL-1β) and apoptosis-related proteins (Bax, caspase-3, caspase-8 and caspase-1) were significantly increased in the EAC bladder (Fig. 9D ∼** 9E**, Supplementary Fig. 6C ∼ 6D, *p* < 0.001), whereas the expression of UPK3A and UPK2 was significantly decreased (Fig. 9G ∼** 9 H**, Supplementary Fig. 6E ∼ 6 F, *p* < 0.05). However, in the bladders of EAC S100A9^−/−^ mice, the expression of the above-mentioned proteins was substantially rescued (Fig. 9D ∼** 9 H**). A similar trend was observed in EAC mice treated with paquinimod (Supplementary Fig. 6C ∼ 6 F). These results suggest that both paquinimod inhibition and the S100A9 knockout can significantly reduce the activation of the TLR4/NF-κB and TLR4/p38 signalling pathways in EAC mice, thereby exerting anti-inflammatory and anti-apoptotic effects.

### Paquinimod Inhibition and the KO of S100A9 significantly improve bladder voiding function in EAC mice

**Fig. 10:**
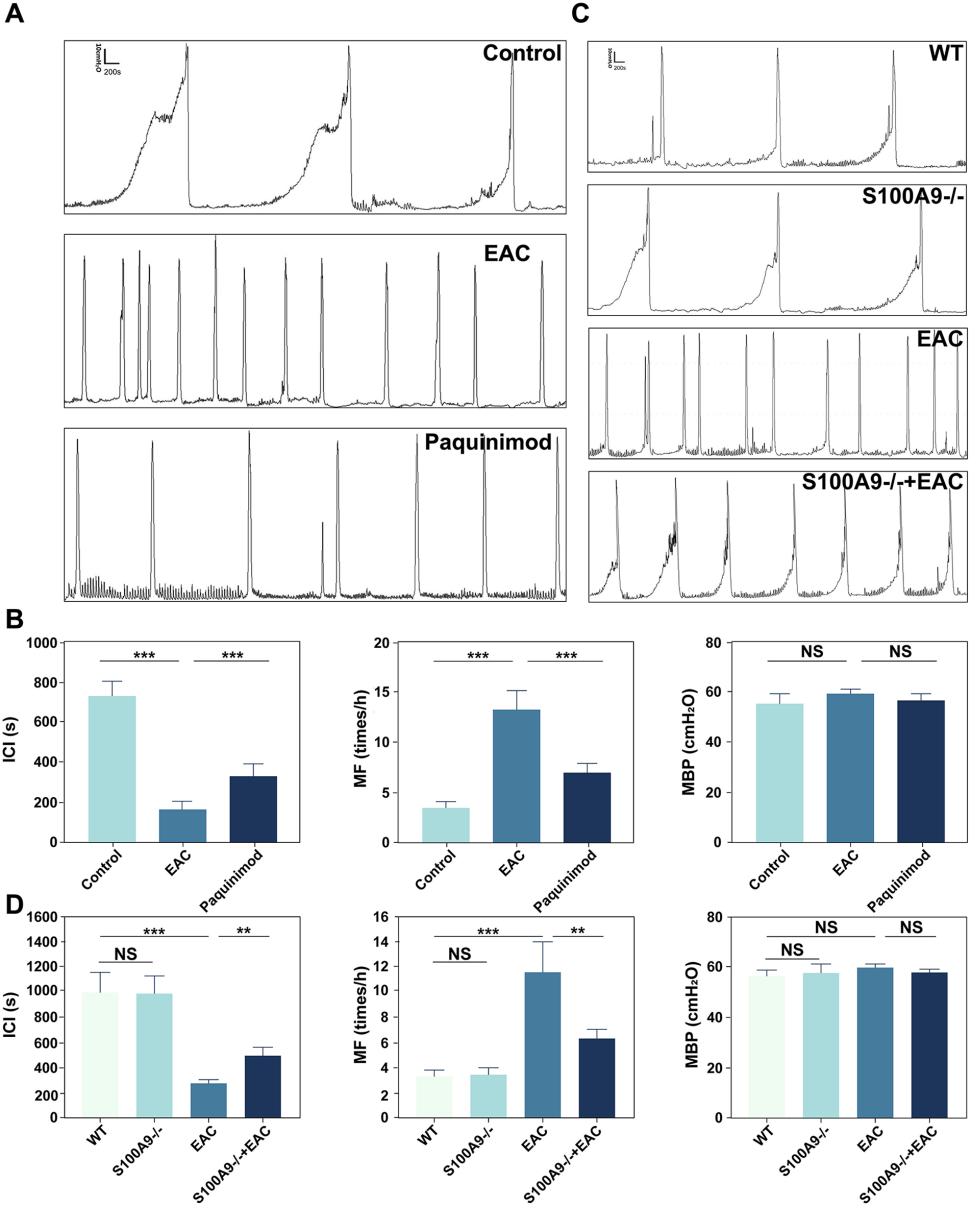
Paquinimod inhibition and S100A9 knockdown significantly improved bladder function in EAC mice (*n* = 6). (**A**) Analysis of cystometry data from control, EAC and paquinimod treatment groups. (**B**) Comparative analysis of micturition frequency (MF), maximum bladder pressure (MBP) and inter-contraction interval (ICI) in the control, EAC and paquinimod treatment groups. (**C**) Analysis of cystometry data of WT, S100A9^−/−^, EAC and S100A9^−/−^ +EAC groups. (**D**) Comparative analysis of MF, MP and ICI in the WT, S100A9^−/−^, EAC and paquinimod treatment groups. NS indicates no difference; *p < 0.05, **p < 0.01, ***p < 0.001

To explore the impact of paquinimod treatment and the S100A9^−/−^ on bladder function in EAC mice, we examined urodynamic performance using cystometry (Fig. 10A ∼** 10D**). No significant difference was found with MBP in the control, WT, S100A9^−/−^, EAC, paquinimod-treated, and S100A9^−/−^ EAC group (Fig. 10B, *p* > 0.05). Compared to the control group, the EAC group showed a significant increase in micturition frequency (MF, Fig. 10B ∼** 10D**, *p* < 0.05) and a significant decrease in the intercontractile interval (ICI, Fig. 10B ∼** 10D**, *p* < 0.05). However, MF was significantly lower (*p* < 0.05) and ICI was significantly longer (*p* < 0.05) in the paquinimod plus EAC group after paquinimod treatment (Fig. 10B and D) (Fig. 10B ∼** 10D**). In addition, compared with the WT and S100A9^−/−^ group (Fig. 10C ∼ 10D), we found a significant increase in MF (*p* < 0.05) and a decrease in ICI (*p* < 0.05) in the EAC group. However, in the S100A9^−/−^ EAC group, the MF was significantly decreased, and the ICI was significantly increased (Fig. 10C ∼ 10D, *p* < 0.05). These results denoted that paquinimod inhibition and S100A9^−/−^ deletion significantly improve bladder urination function in EAC mice.

## Discussion

The aetiology of IC/BPS is multifaceted, and no specific pharmaceutical drugs are currently available [41]. Recent studies indicate that immunological inflammatory responses and regulatory abnormalities prolong and exacerbate IC/BPS [12–17]. S100A8 and S100A9, members of the S100 protein family (also known as the calprotectin protein family), are rapidly released in response to inflammatory stimuli and function as damage-associated molecular patterns (DAMPs) [42]. In immunological and inflammatory disorders, extracellular S100A8 and S100A9 bind TLR4 and activate the innate immune response [18–22]. Studies have implicated S100A8 and S100A9 in the initiation and progression of immunoinflammatory diseases, including autoimmune synovitis, Sjögren’s syndrome, arthritis, systemic sclerosis and systemic lupus erythematosus. Elevated levels of S100A8 and S100A9 have been observed as indicators of disease progression, activity and relapse [18–22, 31, 40, 43]. Both transcriptome sequencing and single-cell sequencing have shown significant differences in the expression of S100A8 and S100A9 in patients with IC/BPS, but their biological function and role remain undetermined [14–16]. In this study, we demonstrated that S100A8 and S100A9 were significantly upregulated in the bladders of IC/BPS patients and EAC mice by proteomic sequencing, single-cell sequencing, bioinformatic analysis, and immunohistochemistry. Interestingly, in contrast to S100A8, S100A9 was overexpressed in the bladders of patients with IC/BPS and EAC mice, and this overexpression was strongly associated with an aberrant immune-inflammatory response. The biological effects of S100A8 and S100A9 predominantly arise through their homodimeric or heterodimeric forms [18–22, 31, 40, 43]. However, studies have shown that S100A9 is an important molecular chaperone for S100A8 and targeted knockdown of S100A9 results in the deletion of both S100A8 and S100A9 [31, 40–43]. Following our research and review of the literature, we identified S100A9 as the principal target molecule for our investigation and analysed its role and function in the bladders of patients with IC/BPS and EAC mice.

The immune-inflammatory response is a crucial defence mechanism of the body [44–46]. A moderate immune-inflammatory response is advantageous for sustaining homeostasis [44–47]. However, an exaggerated or dysregulated immune response can lead to persistent inflammation and irreversible tissue damage [47–49]. In this work, we discovered that the bladders of IC/BPS patients and EAC mice displayed hyperactive immune-inflammatory responses, which involved T cells, macrophages, apoptosis, and other immune-inflammatory signalling activation. In addition, we found active proliferation of immune cells in the bladders of EAC mice. These results suggest that the overactivation and dysregulation of immune inflammatory responses in the bladder of IC/BPS patients and EAC mice are important pathologies. Studies have revealed that macrophages, T cells, and mast cells contribute to IC/BPS pathogenesis [14–17]. In this study, we found that the numbers of bladder macrophages (*P* < 0.01), T cells (*P* < 0.01), and mast cells (*P* < 0.05) were significantly increased in both IC/BPS patients and EAC mice. Furthermore, we observed significant increase in both the number of interactions and the extent of intercellular communication among these immune cells. In particular, the increase in inflammatory regulatory signals received and emitted by macrophages was significant, suggesting that macrophage hyperactivation may play a key role in dysregulated immune-inflammatory responses in IC/BPS and EAC. Studies have indicated that S100A8 and S100A9 are essential for the initiation and progression of immunological inflammation [18–22]. Immune cells, including neutrophils, monocytes, macrophages, as well as cells within localized lesions, are the main sources of S100A8 and S100A9 [50–52]. Under physiological conditions, S100A8 and S100A9 are highly abundant in neutrophils, accounting for approximately 45% of the neutrophil cytoplasm [18]. During immune-inflammatory response, S100A8 and S100A9 are rapidly and massively released, playing a key role in regulating the inflammatory response by attracting inflammatory cells for infiltration and stimulating cytokine secretion [18–22]. In this study, we found that S100A8 and S100A9 were mainly derived from neutrophils. However, bladder macrophages in EAC mice exhibited the most significant increase in the expression of S100A8 and S100A9. Notably, we did not observe intercellular communication between macrophages and neutrophils under normal conditions. However, in EAC, intercellular communication between these two cell types was established. Additionally, the co-occurrence of S100A8 and S100A9 with macrophages in the bladders of IC/BPS patients and EAC mice was confirmed through double-labelling immunofluorescence techniques. These findings suggest that neutrophil-derived S100A8 and S100A9 may induce macrophages to produce inflammatory molecules, thereby promoting inflammatory cascades and contributing to tissue damage in IC/BPS and EAC.

S100A8 and S100A9, endogenous ligands of TLR4, enhance the production of inflammatory cytokines in various immune cells both in vitro and in vivo, thereby prolonging and exacerbating inflammatory responses [51–53]. The overuse of S100A8 and S100A9 exacerbates inflammation and accelerates the infiltration of immune cells, particularly macrophages, creating a self-perpetuating inflammatory cycle that worsens disease progression [18–22]. Inhibition of the downstream signalling pathways of S100A8 and S100A9 significantly reduces pro-inflammatory cytokines and alleviates tissue damage and inflammation [18–22]. In this work, we discovered that upon stimulation with lipopolysaccharide (LPS), primary mouse macrophages may produce and secrete S100A9. Concurrently, exogenous active S100A9 protein induces the release of various inflammatory damage factors, including IL-1β, IL-6, and TNF-α and promote pro-inflammatory activation of primary mouse macrophages. However, the S100A9 inhibitor paquinimod dramatically decreased the expression of inflammatory cytokines in macrophages. Furthermore, research indicates that S100A9 triggers the TLR4/NF-κB and TLR4/P38 signalling pathways, which regulate the production and circulation of various inflammatory cytokines [52–54]. In this study, bioinformatic analysis revealed that inflammatory signalling pathways, including the TLR-MyD88, NF-κB, and P38/MAPK pathways, were activated in both IC/BPS patients and EAC mice. Furthermore, cellular studies provided evidence that S100A9 can initiate the spread of pro-inflammatory agents within macrophages through the TLR4/NF-κB and TLR4-P38 pathways. Additionally, treatment with paquinimod (100 ng/mL) effectively inhibited the activation of the TLR4/NF-κB and TLR4/P38 pathways, thereby preventing the secretion of inflammatory mediators. These findings suggest that S100A9 activates macrophages, leading to the release of inflammatory molecules that contribute to inflammatory injury. To further investigate the role and function of S100A9, animal experiments were conducted.

Research indicates that S100A8 and S100A9 activate the TLR4/p38 and TLR4/NF-κB signalling pathways, leading to the release of IL-1β, IL-6 and TNF-α as well as tissue inflammatory damage [18–22]. In inflammatory conditions, S100A8 and S100A9 contribute to cell death by regulating the synthesis of proteins in the apoptotic cascade and producing reactive oxygen species (ROS) [18, 55, 56]. Oral paquinimod specifically targets and inhibits S100A8 and S100A9, thereby modulating inflammation [18–22]. Paquinimod blocks the binding of S100A8 and S100A9 to the TLR4 receptor, thereby inhibiting the activation of the S100A8- and S100A9-mediated immune-inflammatory signalling pathways, exerting anti-inflammatory and anti-apoptotic effects, and alleviating tissue damage [18–22]. Paquinimod has exhibited promising therapeutic efficacy in many animal models of autoimmune disease. In addition, clinical trials in people diagnosed with autoimmune diseases such as systemic lupus erythematosus and systemic sclerosis have demonstrated the efficacy of paquinimod [18–22, 57, 58]. Urodynamic testing is widely regarded as the preeminent diagnostic tool for assessing bladder function [59, 60]. Decreased bladder capacity, reduced compliance and increased bladder activity are urodynamic features associated with IC/BPS [61, 62]. In several models of cystitis, bladder dysfunction in model mice displayed an augmented urinary frequency and reduced voiding intervals, mirroring the urodynamics observed in IC/BPS patients [63, 64]. In this study, EAC mice were found to have a significantly shorter ICI and a significantly higher MF. However, following paquinimod treatment and the KO of S100A9, MF of EAC mice dramatically decreased, and the ICI was significantly prolonged. Moreover, in the present study, the TLR4/NF-κB and TLR4/p38 signalling pathways were activated in the bladders of EAC mice, accompanied by a significant increase in the expression of inflammatory cytokines (IL-6, TNF-α, and IL-1β) and apoptosis-related proteins (Bax, Caspase-1, Caspase-3, and Caspase-8), along with a marked decrease in the expression of bladder epithelial marker proteins (UPK2 and UPK3A). However, following paquinimod treatment and the KO of S100A9, the activation of the TLR4/NF-κB and TLR4/p38 signalling pathways in EAC mice was significantly inhibited, the expression of inflammatory factors and apoptosis-related proteins was significantly reduced, and the expression of bladder epithelial marker proteins was significantly increased. These results suggest that S100A9 activates the TLR4/NF-κB and TLR4/p38 signalling pathways, resulting in inflammatory bladder injury and impaired bladder function in EAC mice. Paquinimod inhibited and knocked down S100A9 significantly reduced the activation of the TLR4/NF-κB and TLR4/p38 pathways, leading to an improvement in inflammatory bladder injury and bladder function in EAC mice.

Despite the preliminary findings of our research, it is crucial to recognise the inherent limitations of our study, as described below. First, obtaining clinical samples from patients with IC/BPS in China remains challenging due to the limited patient population and the high prevalence of misdiagnosis and underdiagnosis. Consequently, the limited sample size of IC/BPS patients in this study may have influenced the research findings, highlighting the need for larger-scale investigations to validate our results. Second, the findings of this study are inherently biased due to variations in racial, genetic, and environmental factors. Third, the mouse model utilised in this study was established through complete KO of the S100A9 gene, and future research must employ conditional KO mice to validate the findings comprehensively. Fourth, single-cell data and sequencing analysis were not performed on IC/BPS patients in this study. Future investigations should focus on additional sample collection and sequencing analyses to gain a more comprehensive understanding of the underlying mechanisms of IC/BPS. Finally, it should be noted that the research outcomes might be influenced by inherent limitations in the experimental design, research methodology, and the research itself, which could lead to biased results.

## Conclusion

Our study confirmed that S100A8 and S100A9 are important pro-inflammatory pathogenic molecules in IC/BPS and EAC. The activation of the TLR4/NF-κB and TLR4/p38 inflammatory signalling pathways, mediated by S100A8 and S100A9, plays a role in the inflammatory damage of bladder tissue in IC/BPS and EAC. Suppressing S100A9 function, either through inhibition or genetic knockout, significantly alleviate the inflammatory response and mitigates tissue damage in patients with IC/BPS and EAC. These findings imply that S100A9 may hold promise as a potential novel therapeutic target for the treatment and management of IC/BPS. Paquinimod exhibits promising therapeutic properties and holds significant potential in the management of IC/BPS. Our research indicates that paquinimod exerts significant therapeutic effects and could help alleviate IC/BPS symptoms.

## Electronic supplementary material

Below is the link to the electronic supplementary material.

Supplementary Material 1: Supplementary Figure 1. Expression analysis of S100A8 in the bladder of IC/BPS patients and EAC mice. (A-B) Analysis of S100A8 expression in the bladders of IC/BPS patients and EAC mice. (C) ROC curve analysis of S100A8 gene expression in the ulcer and non-ulcer groups of IC/BPS patients. (D) Immunohistochemical analysis of S100A8 expression in EAC mice (x200, n = 6). (E) Immunohistochemical analysis of S100A8 expression in IC/BPS (x200, n = 6). NS indicates no difference; *p<0.05, **p<0.01, ***p<0.001. Supplementary Figure 2. Analysis of bladder cell type and signalling differences between the control and EAC groups. (A) UMAP cluster profiles of bladder cells in control and the EAC groups. (B) UMAP cluster atlas of bladder cell cycle phases. (C) Heatmap showing signalling differences between cells in the EAC group. (D) Heatmap showing signalling differences between cells in the control group. (E-F) Analysis of cell-cell communication between individual cells in the control and EAC groups. Supplementary Figure 3. Cell-cell interaction analysis of bladder cells between control and EAC groups. (A) Comparative analysis of interaction scores between control and EAC groups. (B) Heatmap plot of interaction strengths between different cells in the EAC and control groups. (C) Circle plot showing incremental and decremental changes in signalling between different cell types. (D) Comparison of signalling fluxes between the EAC and control groups. (E) Comparison of overall signalling patterns between the EAC and control groups. (F) Comparative analysis of key macrophage signalling differences between EAC and control groups. (G) Analysis of up- and downregulated signalling received by macrophages in the EAC group. (H) Analysis of up- and downregulated sources from macrophages in the EAC group. Supplementary Figure 4. GO and KEGG analysis and GSEA of single-cell differentially expressed genes in the bladders of EAC mice. (A-D) GO and KEGG pathways enriched from highly expressed genes single-cell sequenced from EAC mice. (E) GSEA analysis of activating pathways from highly expressed genes single-cell sequencing EAC mice. Supplementary Figure 5. Treatment of paquinimod-mediated inhibition of S100A9 on bladder tissue in EAC mice (n = 6). (A) HE staining analysis of paquinimod-mediated inhibition of S100A9 on bladder tissue (x200). (B) Analysis of mast cell infiltration in paquinimod-mediated inhibition of S100A9 on bladder tissues (x200). (C) TUNEL staining analysis of apoptosis in paquinimod-mediated inhibition of S100A9 on bladder tissues (x200). (D) Immunohistochemical analysis of macrophage marker F4/80 in paquinimod-mediated inhibition of S100A9 (x200). (E) Immunohistochemical analysis of T cell marker CD4 on paquinimod-mediated inhibition of S100A9 (x200). (F) Immunohistochemical analysis of S100A9 on paquinimod-mediated inhibition of S100A9 (x200). NS indicates no difference; *p<0.05, **p<0.01, ***p<0.001. Supplementary Figure 6. Paquinimod-mediated inhibition of S100A9 significantly reduced TLR4/NF-?B and TLR4/p38 signalling pathway activation and decreased inflammation and apoptosis-related protein expression in EAC mice (n = 6). (A and B) Western blot analysis of TLR4/NF-?B and TLR4/p38 signalling pathway proteins in paquinimod-mediated inhibition of S100A9 in EAC mice (C-D) Western blot analysis of inflammation-related proteins (IL-6, IL-1?, and TNF-?) in paquinimod-mediated inhibition of S100A9 in EAC mice. (E-F) Western blot analysis of apoptosis-related proteins (Bax, caspase-3, caspase-8, and caspase-1) and epithelial damage marker proteins (UPK3A and UPK2) in paquinimod-mediated inhibition of S100A9 in EAC mice. NS indicates no difference; *p<0.05, **p<0.01, ***p<0.001.

## Data Availability

No datasets were generated or analysed during the current study.
