# Supplementary material for: S100A9 as a potential novel target for experimental autoimmune cystitis and interstitial cystitis/bladder pain syndrome
Source: Biomark Res. 2025 May 9;13:72. doi: 10.1186/s40364-025-00763-5 (PMC12065242; doi:10.1186/s40364-025-00763-5)
Supplement: Supplementary file 1 — Supplementary Material 1: Supplementary Figure 1. Expression analysis of S100A8 in the bladder of IC/BPS patients and EAC mice. (A-B) Analysis of S100A8 expression in the bladders of IC/BPS patients and EAC mice. (C) ROC curve analysis of S100A8 gene expression in the ulcer and non-ulcer groups of IC/BPS patients. (D) Immunohistochemical analysis of S100A8 expression in EAC mice (x200, n = 6). (E) Immunohistochemical analysis of S100A8 expression in IC/BPS (x200, n = 6). NS indicates no difference; *p<0.05, **p<0.01, ***p<0.001. Supplementary Figure 2. Analysis of bladder cell type and signalling differences between the control and EAC groups. (A) UMAP cluster profiles of bladder cells in control and the EAC groups. (B) UMAP cluster atlas of bladder cell cycle phases. (C) Heatmap showing signalling differences between cells in the EAC group. (D) Heatmap showing signalling differences between cells in the control group. (E-F) Analysis of cell-cell communication between individual cells in the control and EAC groups. Supplementary Figure 3. Cell-cell interaction analysis of bladder cells between control and EAC groups. (A) Comparative analysis of interaction scores between control and EAC groups. (B) Heatmap plot of interaction strengths between different cells in the EAC and control groups. (C) Circle plot showing incremental and decremental changes in signalling between different cell types. (D) Comparison of signalling fluxes between the EAC and control groups. (E) Comparison of overall signalling patterns between the EAC and control groups. (F) Comparative analysis of key macrophage signalling differences between EAC and control groups. (G) Analysis of up- and downregulated signalling received by macrophages in the EAC group. (H) Analysis of up- and downregulated sources from macrophages in the EAC group. Supplementary Figure 4. GO and KEGG analysis and GSEA of single-cell differentially expressed genes in the bladders of EAC mice. (A-D) GO and KEGG pathways enric [file 40364_2025_763_MOESM1_ESM.docx]

**Supplementary Table 1 Histology Score Based on Inflammation.**

| Score | Histological Characteristics |
| --- | --- |
| 0 | Morphologically unremarkable with no or very minimal inflammation or epithelial changes. |
| 1 | Minimal inflammatory infiltrate composed of occasional neutrophils or lymphocytes within the lamina propria in the absence of inflammation in the muscularis propria, or significant edema, hemorrhage or urothelial changes. |
| 2 | Minimal to mild inflammatory infiltrate within the lamina propria with scattered neutrophils or lymphocytes, accompanied by mild edema or hemorrhage, but in the absence of inflammation in the muscularis propria or significant urothelial changes. |
| 3 | Mild or mild to moderate inflammatory infiltrate in the lamina propria and focal extension of the inflammation into the muscularis propria. |
| 4 | Moderate inflammation with scattered to frequent neutrophils and lymphocytes in both the lamina propria and muscularis propria. |
| 5 | Severe inflammation in the lamina propria and muscularis propria in association with other significant findings, such as urothelial ulceration, severe edema, hemorrhage and fibrin deposition. |


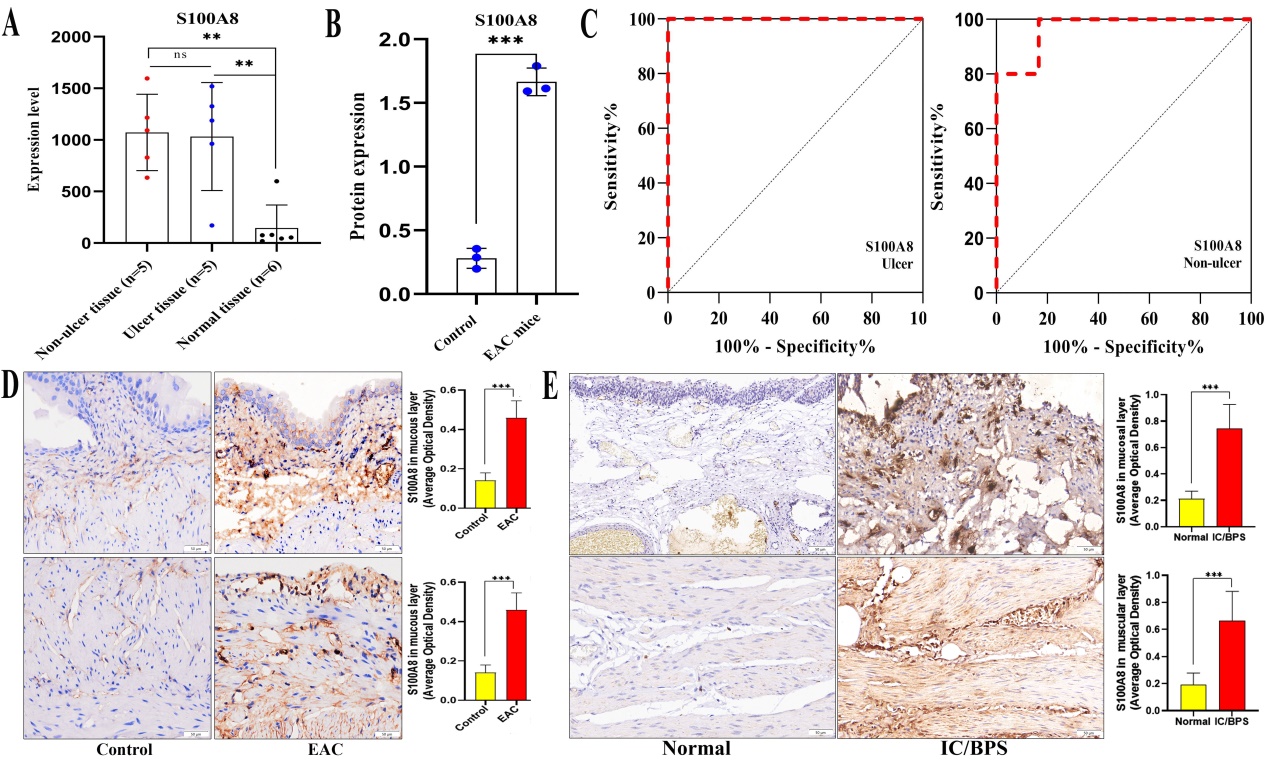


**Supplementary Fig. 1. Expression analysis of S100A8 in the bladder of IC/BPS patients and EAC mice.**

(A-B) Analysis of S100A8 expression in the bladders of IC/BPS patients and EAC mice. (C) ROC curve analysis of S100A8 gene expression in the ulcer and non-ulcer groups of IC/BPS patients. (D) Immunohistochemical analysis of S100A8 expression in EAC mice (×200, n = 6). (E) Immunohistochemical analysis of S100A8 expression in IC/BPS (×200, n = 6). NS indicates no difference; *p < 0.05, **p < 0.01, ***p < 0.001.

**Supplementary Fig. 2. Analysis of bladder cell type and signalling differences between the control and EAC groups.**

(A) UMAP cluster profiles of bladder cells in control and the EAC groups. (B) UMAP cluster atlas of bladder cell cycle phases. (C) Heatmap showing signalling differences between cells in the EAC group. (D) Heatmap showing signalling differences between cells in the control group. (E-F) Analysis of cell-cell communication between individual cells in the control and EAC groups.


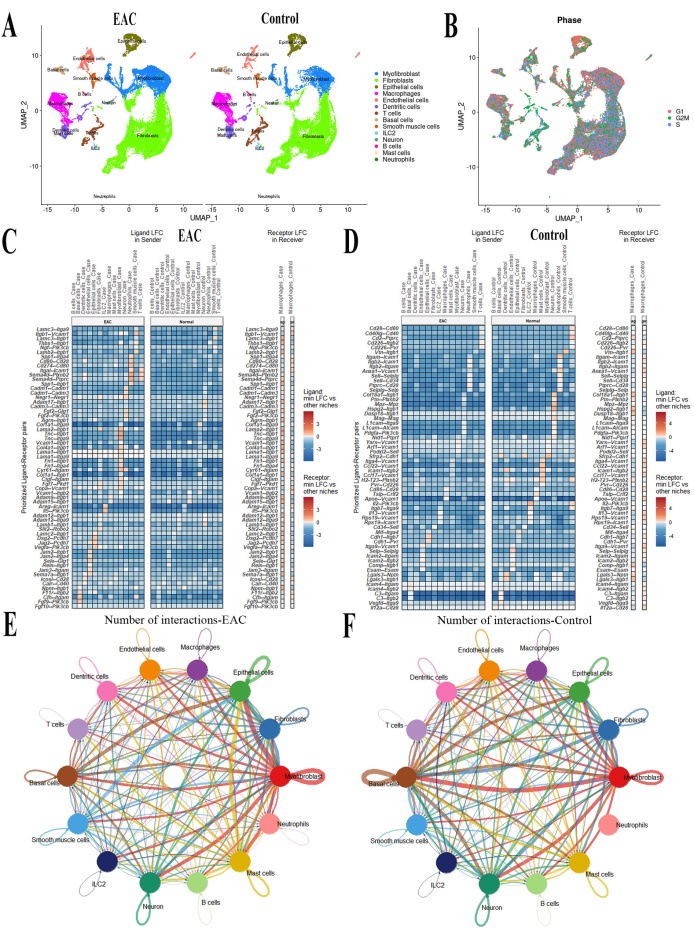


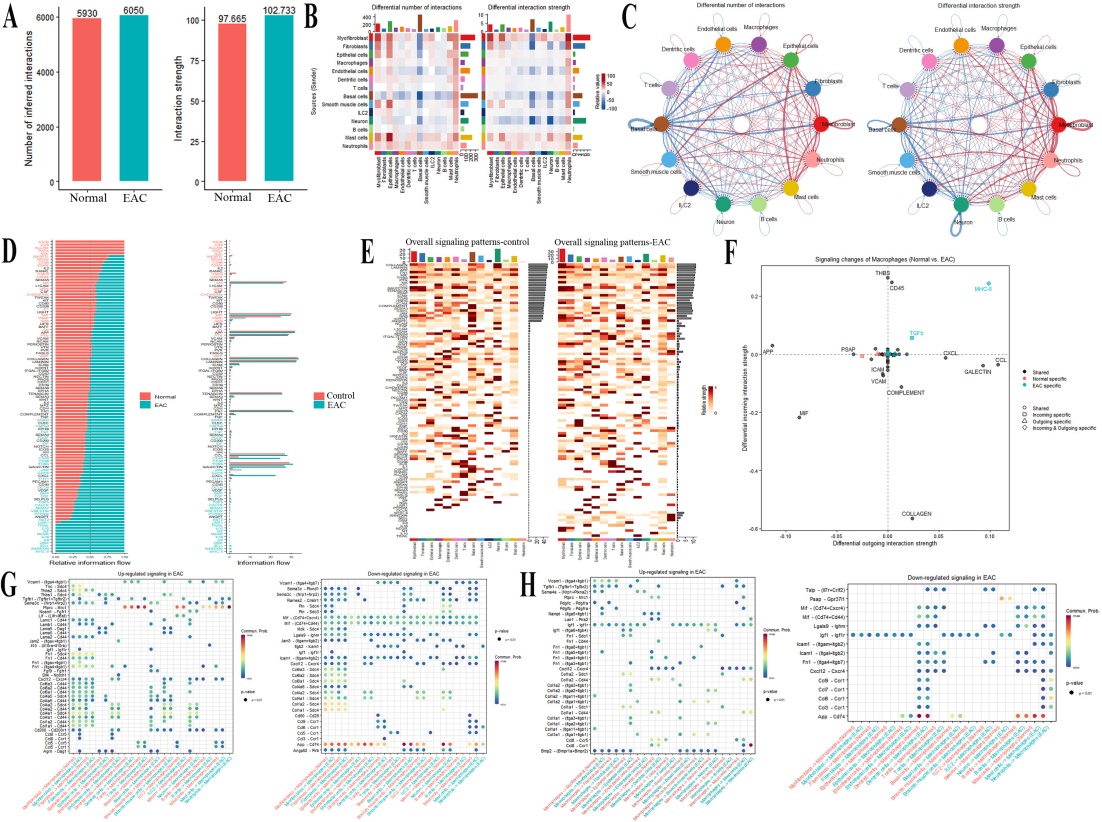


**Supplementary Fig. 3. Cell-cell interaction analysis of bladder cells between control and EAC groups.**

(A) Comparative analysis of interaction scores between control and EAC groups. (B) Heatmap plot of interaction strengths between different cells in the EAC and control groups. (C) Circle plot showing incremental and decremental changes in signalling between different cell types. (D) Comparison of signalling fluxes between the EAC and control groups. (E) Comparison of overall signalling patterns between the EAC and control groups. (F) Comparative analysis of key macrophage signalling differences between EAC and control groups. (G) Analysis of up- and downregulated signalling received by macrophages in the EAC group. (H) Analysis of up- and downregulated sources from macrophages in the EAC group.


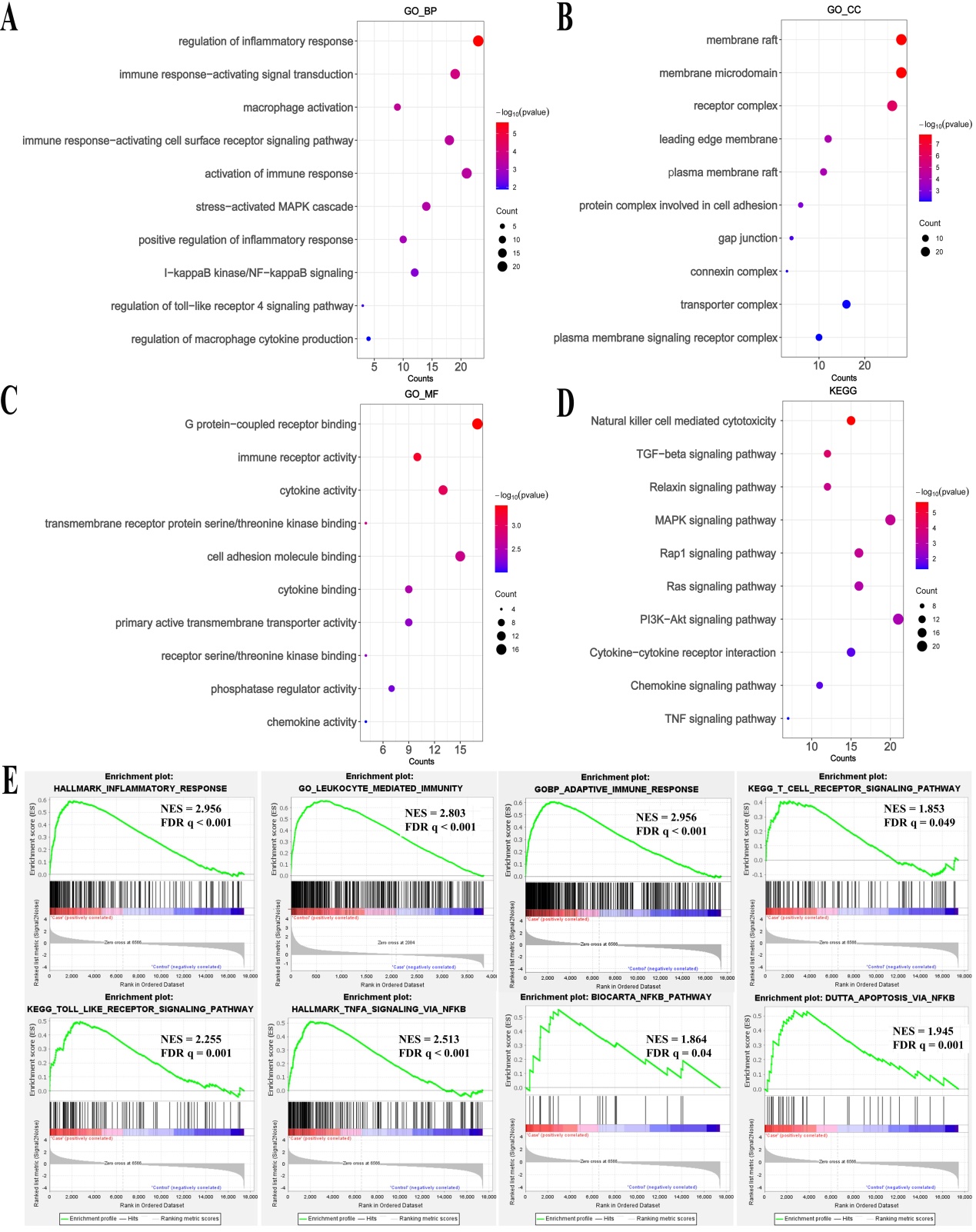


**Supplementary Fig. 4. GO and KEGG analysis and GSEA of single-cell differentially expressed genes in the bladders of EAC mice.**

1. D) GO and KEGG pathways enriched from highly expressed genes single-cell sequenced from EAC mice. (E) GSEA analysis of activating pathways from highly expressed genes single-cell sequencing EAC mice.


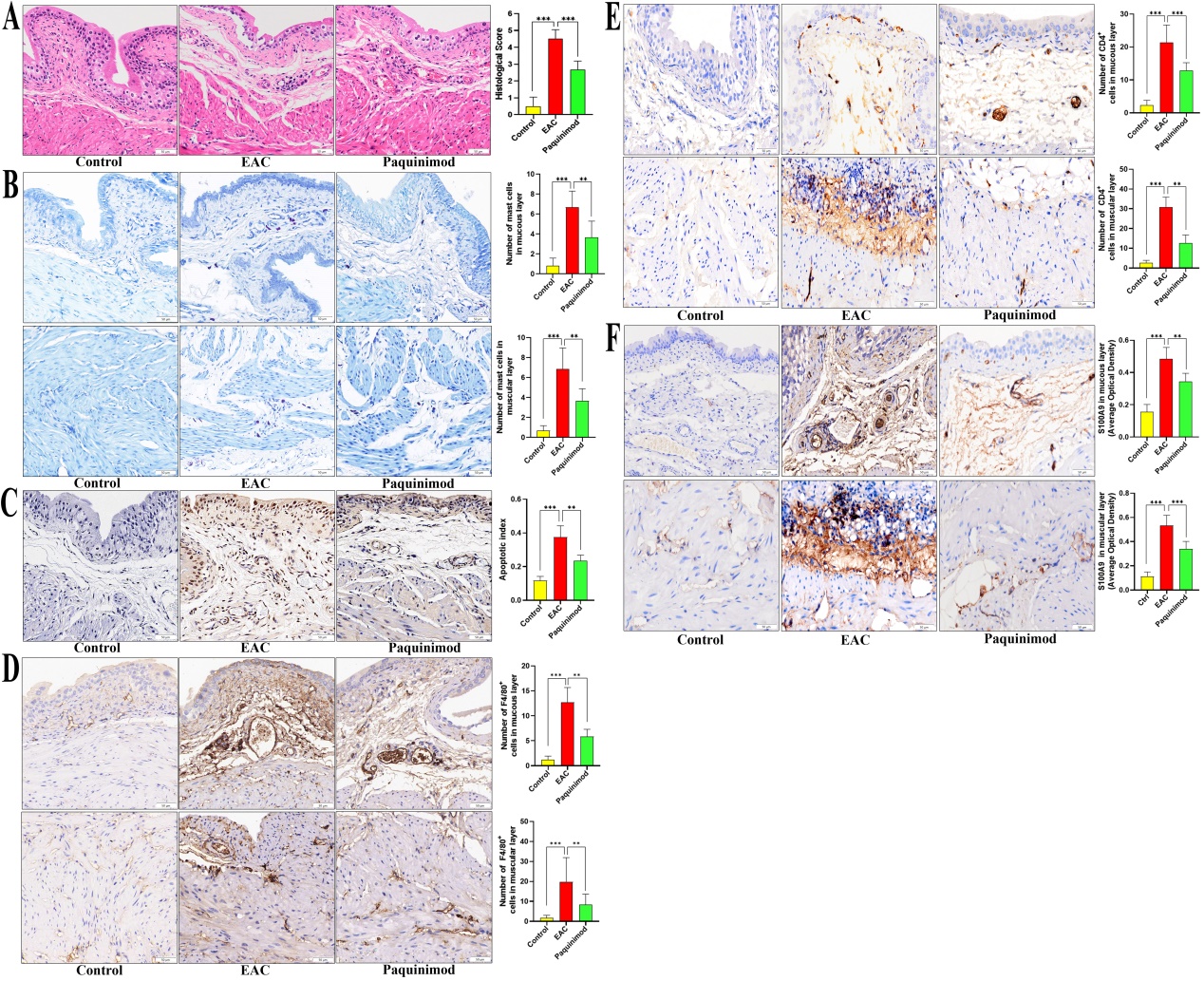


**Supplementary Fig. 5.** Treatment of paquinimod-mediated inhibition of S100A9 on bladder tissue in EAC mice (n = 6).

(A) HE staining analysis of paquinimod-mediated inhibition of S100A9 on bladder tissue (×200). (B) Analysis of mast cell infiltration in paquinimod-mediated inhibition of S100A9 on bladder tissues (×200). (C) TUNEL staining analysis of apoptosis in paquinimod-mediated inhibition of S100A9 on bladder tissues (×200). (D) Immunohistochemical analysis of macrophage marker F4/80 in paquinimod-mediated inhibition of S100A9 (×200). (E) Immunohistochemical analysis of T cell marker CD4 on paquinimod-mediated inhibition of S100A9 (×200). (F) Immunohistochemical analysis of S100A9 on paquinimod-mediated inhibition of S100A9 (×200). NS indicates no difference; *p < 0.05, **p < 0.01, ***p < 0.001.


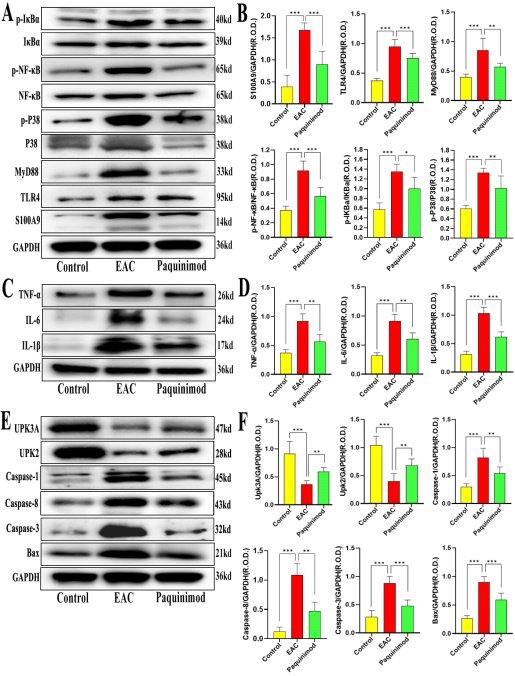


**Supplementary Fig. 6. Paquinimod-mediated inhibition of S100A9 significantly reduced TLR4/NF-κB and TLR4/p38 signalling pathway activation and decreased inflammation and apoptosis-related protein expression in EAC mice (n = 6).**

(A and B) Western blot analysis of TLR4/NF-κB and TLR4/p38 signalling pathway proteins in paquinimod-mediated inhibition of S100A9 in EAC mice (C-D) Western blot analysis of inflammation-related proteins (IL-6, IL-1β, and TNF-α) in paquinimod-mediated inhibition of S100A9 in EAC mice. (E-F) Western blot analysis of apoptosis-related proteins (Bax, caspase-3, caspase-8, and caspase-1) and epithelial damage marker proteins (UPK3A and UPK2) in paquinimod-mediated inhibition of S100A9 in EAC mice. NS indicates no difference; *p < 0.05, **p < 0.01, ***p < 0.001.
